# DC Magnetron-Generated Reactive Glow Discharge Plasma Activation of Acid-Etched Titanium for Superhydrophilic TiO_2_‑Rich Surfaces

**DOI:** 10.1021/acsomega.6c06100

**Published:** 2026-10-21

**Authors:** Mickael Melo Linkiewicz, Angelika von Schmude, Matheus H. L. de Lima, Anderson R. Carvalho, Marcel F. Kunrath, Marcus Seferin, Roberto Hübler

**Affiliations:** † Materials and Nanoscience Laboratory, Interdisciplinary Center for Nanoscience and Micro-Nanotechnology (NanoPUCRS), 28102 Polytechnic School, PUCRS, 91530-001, Porto Alegre, RS, Brazil; ‡ Institute of Physics, UFRGS, 91501-970, Porto Alegre, RS, Brazil; § Interdisciplinary Center for Nanoscience and Micro-Nanotechnology (NanoPUCRS), 28102 Polytechnic School, PUCRS, 91530-001, Porto Alegre, Rio Grande do Sul, Brazil; ∥ Chemometrics and Analytical Instrumentation Laboratory, Institute of Chemistry, UFRGS, 91501-970, Porto Alegre, Rio Grande do Sul, Brazil; ⊥ Department of Dentistry, School of Health and Life Sciences, PUCRS, 90619-900, Porto Alegre, Rio Grande do Sul, Brazil; # Department of Biomaterials, Institute of Clinical Sciences, Sahlgrenska Academy at the University of Gothenburg, 40530, Göteborg, Sweden; ∇ Industrial Chemistry Laboratory, 28102 Polytechnic School, PUCRS, 90619-900, Porto Alegre, RS, Brazil

## Abstract

This study addresses the need for controllable physicochemical activation routes for titanium surfaces by developing a hybrid treatment that combines hydrofluoric acid (HF) microtexturing with reactive glow discharge plasma (GDP) generated in a DC magnetron sputtering (DC-MS) system, applied to commercially pure grade 1 titanium. Sequential acid etching induced controlled microstructural roughening, followed by a 1 min plasma exposure in air at 6 × 10^–1^ Pa to modify the near-surface oxide chemistry while preserving the etched morphology. Acid treatment preferentially dissolved the α-Ti matrix along grain boundaries, producing band-like corrosion grooves and hierarchical microtexturing, whereas plasma activation induced localized oxidation and produced a superhydrophilic response. Eight activation cycles over 150 days of ambient storage repeatedly restored the hydrophilic state, which persisted approximately 16 h per cycle. Raw X-ray diffraction (XRD) and grazing-incidence XRD patterns remained dominated by α-Ti, with no resolved reflections attributable to crystalline TiO_2_. X-ray photoelectron spectroscopy indicated the formation of a TiO_2_-rich surface after plasma activation, accounting for 100% of the fitted Ti 2p area, with no detectable F 1s signal. Adsorbed oxygen-containing species accounted for 4.7%, compared with 4.3% for the etched and 17.4% for the cleaned control. Because biological responses were not evaluated, these physicochemical changes should not be interpreted as direct evidence of improved protein adsorption, cellular behavior, bacterial resistance, or in vivo performance. This approach establishes DC-MS-generated GDP activation as a rapid route for titanium surface activation and advanced surface-engineering applications.

## Graphical abstract

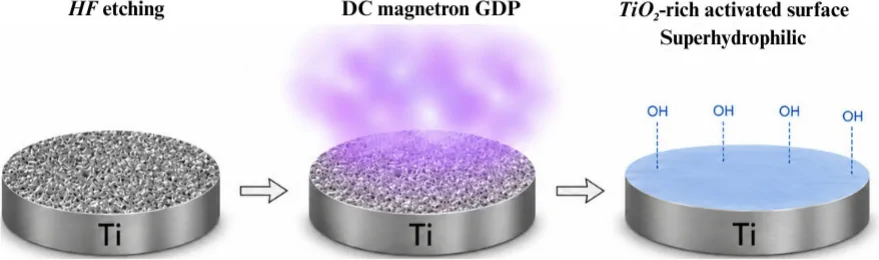

## Introduction

Titanium (*Ti*) and its alloys remain the benchmark materials for load-bearing biomedical and engineering interfaces due to their corrosion resistance, mechanical reliability, and ability to establish stable interfacial bonding with surrounding environments.^1,2^ Interfacial performance is primarily governed by surface-dependent phenomena, in which micro- and nanoscale topography, oxide chemistry, surface energy, and wettability collectively regulate physicochemical interactions and subsequent functional responses.^3,4^ In implant-relevant contexts, this process — often described as osseointegration — depends strongly on the structural and chemical state of the titanium oxide layer, which modulates protein adsorption, cellular attachment, and early interfacial stabilization.^1,3^ Consequently, precise engineering of titanium surface properties has become a central strategy for controlling functional interface performance across biomedical and advanced materials applications.^5–7^

To enhance osteoconductivity and interfacial performance, subtractive surface modification techniques such as sandblasting and acid etching (SLA) have become industrially established approaches for generating micro- and nanotextured titanium surfaces.^8^ Among these, hydrofluoric acid (*HF*) etching is particularly effective at dissolving the native passive oxide layer and producing nanotopographies that increase the surface area and promote enhanced physicochemical reactivity.^9–12^ Despite these advantages, important limitations remain. Chemical processing may introduce residual contaminants, including fluorine-derived species, and surfaces may exhibit hydrophobic behavior due to the rapid adsorption of atmospheric hydrocarbons, even when favorable roughness is achieved.^8,9,13^ This process, commonly termed the biological aging of titanium, leads to progressive reductions in surface free energy and interfacial activity.^14,15^ The formation of hydrocarbon layers can delay protein adsorption and impair early stage interfacial interactions critical for functional surface performance.^16,17^

Overcoming biological aging requires surface activation to restore the hydrophilicity. Commercial solutions such as wet storage (e.g., *SLActive*) effectively prevent contamination but involve significant manufacturing and packaging complexities.^18,19^ Alternatively, chair-side treatments such as ultraviolet (UV) photofunctionalization have been proposed to decompose surface hydrocarbons and induce superhydrophilicity.^20–22^ However, UV treatment often requires long exposure times (>3 h) to achieve optimal results, thereby limiting its clinical practicality.^20,21,23^

Nonthermal plasma technologies have emerged as rapid and efficient alternatives for surface activation. Reported titanium configurations are dominated by atmospheric-pressure sources, operated as nonthermal atmospheric-pressure plasma (NTAP) or cold atmospheric plasma (CAP),^24–31^ with fewer studies employing low-pressure and vacuum-assisted discharges,^32,33^ chair-side device implementations,^34,35^ and comparative evaluations of plasma-conditioned surfaces.^23,36^ Plasma treatment generates reactive species, including ions, electrons, and radicals, that interact with the surface, removing organic contaminants and modifying oxide chemistry and surface polarity, thereby enhancing wettability^37^ and potentially influencing subsequent biological interactions.^38^ While various plasma setups exist (e.g., atmospheric pressure plasma jets and dielectric barrier discharge),^24–28,39,40^ there is growing interest in leveraging existing industrial deposition infrastructure for surface activation.^41–43^

Direct current magnetron sputtering (DC-MS) systems are widely used for thin film deposition.^44–46^ However, using a DC-MS source not for deposition but to generate reactive glow discharge plasma (GDP) from air admitted into a low-pressure chamber remains of interest for cost-effective surface activation. This configuration exploits the magnetron’s magnetic confinement to sustain a stable glow discharge while avoiding intentional film deposition, thereby enabling modification of the near-surface titanium oxide chemistry. Across the configurations surveyed above, no reported study combines an air-fed DC-MS-generated GDP operated at approximately 6 × 10^–1^ Pa with repeated reactivation cycles monitored over 150 days of ambient storage.

This study proposes a process-oriented surface engineering approach based on a hybrid activation protocol that combines hydrofluoric acid etching with a reactive GDP generated by a DC-MS source operating under an air-dominant atmosphere at approximately 6 × 10^–1^ Pa, 22 W and 440 V for 1 min. Rather than employing the magnetron system for film deposition, the plasma is used as a rapid activation tool to modify the physicochemical properties of grade 1 titanium surfaces. The effects of this treatment are systematically investigated in terms of surface morphology, roughness, wettability, crystalline structure, and surface chemistry. Additionally, the temporal stability of the plasma-induced hydrophilic state and the effectiveness of repeated plasma reactivation over eight cycles spanning 150 days during long-term aging are assessed. By focusing exclusively on surface and interface engineering, this work establishes a robust physicochemical foundation for future application-driven investigations while highlighting the potential of DC magnetron sources for reactive GDP activation.

## Materials and Methods

### Titanium Sample Preparation

A commercially pure grade 1 titanium sheet (**ASTM F67**)^47^ with a thickness of 0.60 mm was laser-cut (JL Laser JL Soldas, Novo Hamburgo, RS, Brazil) into disk-shaped specimens with a diameter of 8.00 mm. The samples were subjected to different surface modification protocols and classified into three experimental groups: control cleaning (1CC), acid etching (2AE), and reactive plasma activation (3RP).

#### Control Cleaning (1CC)

The control cleaning protocol was designed to remove contaminants generated during the laser cutting process. The titanium disks were subjected to a multistep ultrasonic cleaning sequence as follows: (I) immersion in deionized (DI) water containing 0.05% (v/v) neutral detergent under ultrasonication at 40 kHz for 5 min; (II) rinsing with DI water; (III) ultrasonic cleaning in DI water for an additional 5 min; (IV) drying under a pressurized nitrogen (*N*_2_) gas stream; (V) immersion in analytical-grade isopropyl alcohol under ultrasonication for 5 min; and (VI) final drying with a *N*_2_ gas stream. After being cleaned, the samples were stored at room temperature in a dust-free environment until further processing.

#### Acid Etching (2AE)

Treatment was performed by immersing the titanium disks into a 6% (v/v) aqueous hydrofluoric acid (*HF*), prepared by diluting a 48% stock solution (*EMSURE*, *Merck*). The etching was conducted in a polypropylene container under ultrasonic agitation (40 kHz) for 2 min. Subsequently, the samples underwent the same post-treatment cleaning protocol described for the 1CC group (Steps II–VI).

#### Safety Statement

Hydrofluoric acid is highly corrosive and toxic and can cause severe local and systemic injuries. All *HF* handling and dilution steps were performed in a chemical fume hood using *HF*-compatible polypropylene vessels and appropriate personal protective equipment, including chemical-resistant gloves, laboratory coats, and face protection, following institutional chemical safety procedures.

#### Reactive Plasma Activation (3RP)

Plasma activation was conducted in a custom-built vacuum chamber equipped with a turbomolecular pump, a mechanical backing pump, and a DC power supply. The system incorporates a DC magnetron sputtering source consisting of a high-purity titanium (target) cathode and a concentric ring anode, which confines the plasma discharge to the central region of the chamber (Figure 1A). Prior to plasma exposure, samples previously subjected to the 2AE treatment were placed directly onto the cathode surface (Figure 1B). The chamber was evacuated to a base pressure of approximately 8 × 10^–4^ Pa, after which ambient air was introduced through a needle valve until a working pressure of approximately 6 × 10^–1^ Pa was reached. A toroidal glow discharge was ignited by applying a power of 22 W at approximately 440 V and the discharge was maintained for 1 min (Figure 1C). After treatment, the samples were stored at room temperature until characterization.

**Figure 1:**
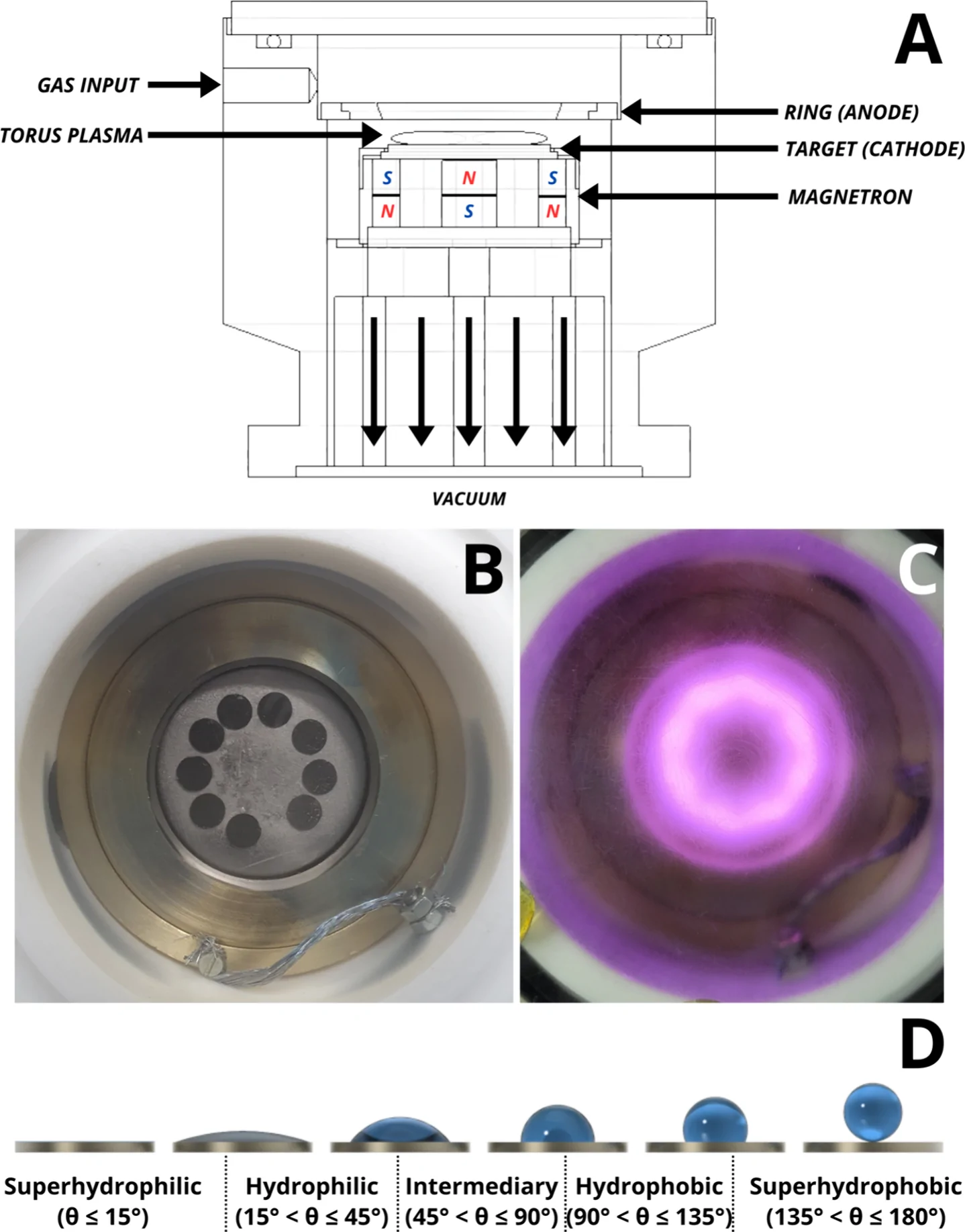
Experimental setup for plasma surface treatment and wettability assessment. (A) Schematic cross-section of the DC magnetron sputtering system showing the titanium cathode, ring anode, and magnetron assembly. (B) Top view of the vacuum chamber with samples positioned on the target before plasma exposure. (C) Photograph of the glow discharge plasma generated over the sample surface during treatment. (D) Water contact angle scale used for wettability classification, adapted from **ASTM D7334**. The hydrophilic, intermediate, and hydrophobic bands are defined by the standard, whereas the superhydrophilic and superhydrophobic bands were introduced in the present work.

For XPS analysis, the same titanium disk was sequentially evaluated under 1CC, 2AE, 3RP, and 3RP-A conditions. Before each XPS acquisition, the specimen was introduced into the XPS system and maintained under vacuum for approximately 48 h. Following the 1CC analysis, the disk was removed, subjected to *HF* etching and the corresponding post-treatment cleaning procedure, and reintroduced for analysis as 2AE. The same disk was subsequently cleaned, subjected to plasma activation, immediately transferred to the XPS facility, and analyzed as 3RP after approximately 48 h under vacuum. After the 3RP acquisition, the disk was removed, cleaned using the control-cleaning protocol, stored in a desiccator, and subsequently reintroduced into the XPS system for a second postplasma analysis, designated 3RP-A, after an additional approximately 48 h under vacuum. The aged reactive plasma condition (3RP-A) represents the same plasma-activated disk after an intervening cleaning, desiccator storage, and vacuum equilibration sequence. Therefore, 3RP-A should not be interpreted as a pure atmospheric aging condition.

### Surface Characterization

#### Surface Morphology

Surface morphology was examined using field emission scanning electron microscopy (FESEM; Inspect F50, FEI Company, Prague, Czech Republic). Morphological screening was performed on 61 disks from the 1CC/2AE processing sequence and 21 disks from the 3RP group. Representative micrographs were selected to illustrate the characteristic surface features induced by each treatment. Micrographs were acquired at accelerating voltages of 10 and 20 kV, with magnifications ranging from 100× to 100,000×.

#### Surface Roughness

Quantitative surface roughness measurements were performed using a contact profilometer (Surftest SJ-201P, Mitutoyo, Kawasaki, Japan), in accordance with **ISO 4287** and **ISO 4288** standards. Surface roughness was evaluated on n = 60 independent disks for 1CC, n = 60 independent disks for 2AE, and n = 20 independent disks for 3RP. A cutoff length (λ_*C*_) of 0.8 mm and a short-wavelength filter (λ_*s*_) of 2.5 μm were applied, with an evaluation length comprising three consecutive sampling lengths.^48,49^

#### Surface Hydrophilicity

Surface wettability was assessed via static contact angle measurements using the sessile drop method in accordance with **ASTM D7334**, whereby the angle measured immediately after deposition of a maximum-volume sessile drop is defined as the advancing contact angle.^50^ Measurements were performed on n = 60 independent disks for 1CC, n = 60 independent disks for 2AE, and n = 20 independent disks for 3RP. The relative wetting ratio (*r*) was calculated from the same data set. Short-term wettability stability was evaluated using n = 3 independent 3RP disks. A goniometer (SEO Phoenix 300, Kosek Dong, South Korea) was used to dispense a 15 ± 5 μL droplets of deionized water onto the center of each sample surface, away from the laser-cut perimeter. Images were captured within 30 s of droplet deposition using the associated software (Surfaceware 9), and the contact angle of each specimen was taken as the mean of the one drop-edge angles. All measurements were conducted under controlled ambient conditions (23 ± 2 °C and relative humidity ≥ 50%), as prescribed by the standard.

A single droplet was deposited per specimen. On the etched and plasma-activated surfaces, the droplet spreads over essentially the entire disk, leaving no unwetted area for a second deposition. Moreover, because recleaning a wetted specimen alters its surface state — an effect explicitly acknowledged in the standard — a repeated measurement would not represent the original surface condition. Replication was therefore performed at the specimen level rather than at the droplet level, consistent with the treatment of the titanium disk as the independent experimental unit. The remaining deviations from the standard were limited to the use of deionized water instead of Type II reagent water and a droplet-volume tolerance of ± 5 μL rather than of ± 0.1 μL. The rough and highly wettable character of the 2AE and 3RP surfaces, identified in the standard as a potential interference, was addressed by acquiring all images within the 30 s window.

Wettability was classified according to the scale shown in Figure 1D, adapted from **ASTM D7334**.^50^ The hydrophilic (θ < 45°), intermediate (45° ≤ θ ≤ 90°), and hydrophobic (θ > 90°) bands are those defined in the standard. Two additional bands were introduced in the present work to characterize the extremes of the scale: superhydrophilic, defined as θ ≤ 15°, corresponding to the midpoint of the 10–20° range identified in the standard as indicative of excellent wetting; and superhydrophobic, defined as θ > 135°, corresponding to the midpoint of the hydrophobic domain between 90° and 180°.

Long-term plasma reactivation stability was evaluated using n = 20 *HF*-etched and plasma-activated titanium disks subjected to eight activation cycles, consisting of the initial plasma activation followed by seven reactivations applied after ambient storage intervals of 1, 2, 7, 14, 21, 28, 35, and 42 days, giving cumulative storage times of 1, 3, 10, 24, 45, 73, 108, and 150 days. Static water contact angles were measured immediately after each activation or reactivation and after 24 h of ambient aging by using the sessile drop protocol described above.

Short-term wettability stability was evaluated on n = 3 independent 3RP disks stored in ambient air without reactivation and measured at 0, 3, 6, 9, 12, 16, 19, 22, 25, and 28 h after activation. The same three disks were measured at each time point; therefore, these data constitute repeated measures and were evaluated descriptively. After each measurement, the disks were dried under a stream of dry nitrogen to remove residual water before being returned to ambient storage. This drying step was not applied to the disks of the long-term reactivation series.

#### Surface Crystallinity

Crystalline-phase analysis was performed by X-ray diffraction (XRD) using a Shimadzu XRD-7000 diffractometer (Kyoto, Japan) equipped with a *Cu Kα* radiation source (λ = 1.5406 Å). XRD and GIXRD analyses were performed on one sequentially treated disk (n = 1), which was analyzed after 1CC, 2AE, and 3RP. This within-specimen design was adopted to reduce sample-to-sample variability associated with the initial crystallographic texture and surface heterogeneity of the titanium disks. The system operated at 40 kV and 30 mA, and diffraction patterns were collected over a *2θ* range of 20–50° using conventional Bragg–Brentano and grazing-incidence configurations, the latter at a fixed incidence angle of 1°. The *2θ* range was restricted to 20–50° for instrumental reasons. Raw diffraction patterns were used for qualitative comparison among the sequential treatment conditions. No background subtraction, normalization, low-angle halo integration, quantitative phase-fraction analysis, or quantitative crystallographic texture analysis was performed. The patterns were vertically offset only for graphical clarity.

#### Surface Chemistry

Surface chemical composition was analyzed by X-ray photoelectron spectroscopy (XPS) using a Scienta Omicron spectrometer (NanoTechnology GmbH, Taunusstein, Germany) equipped with a hemispherical analyzer (Sphera) and a monochromatic *Al Kα* X-ray source (1486.6 eV). XPS analysis was performed on one sequentially treated disk (n = 1) analyzed after 1CC, 2AE, 3RP, and 3RP-A. This sequential design was adopted to minimize sample-to-sample variability in the comparison of surface chemical evolution. Spectral deconvolution and quantitative analyses were performed using CasaXPS and Origin software. Because *O–N* and *O–H* species have overlapping *O 1s* binding-energy ranges near 531.5 eV, they were represented by a single unresolved *O–N/O–H* component. Treatment-dependent interpretation of this component was based on complementary *N 1s* and *Ti 2p* evidence and was not treated as a direct spectral separation of *O–N* and *O–H* species.

#### Statistical Analysis

Quantitative data are presented as mean ± standard deviation unless otherwise stated. For surface roughness, static contact angle, *Wenzel* roughness factor, and short-term wettability stability analyses, the titanium disk was considered the independent experimental unit. Data normality was assessed using *Anderson–Darling*, *D’Agostino–Pearson*, *Shapiro–Wilk*, and *Kolmogorov–Smirnov* tests. Because most data sets did not follow a normal distribution, nonparametric statistical analyses were performed using the *Kruskal–Wallis* test followed by *Dunn’s* multiple-comparison post hoc test to identify significant differences between groups. XRD/GIXRD and XPS measurements were performed on a single sequentially treated disk and were therefore evaluated descriptively; no inferential statistical analyses were applied to these data sets. Because the *O 1s* contribution near 531.5 eV was unresolved and the XPS and contact-angle measurements were obtained from different experimental units and replication schemes, no inferential correlation between XPS peak-area contributions and wettability was performed. Statistical analyses and graphical outputs were performed using GraphPad Prism software version 8.4.3 (GraphPad Prism Software, San Diego, CA, USA).

## Results and Discussion

### Surface Characterization of Plasma-Activated Titanium Samples

#### Surface Morphology

Figure 2 presents FESEM images acquired in secondary-electron (SE) and backscattered-electron (BE) modes at 20 kV. The 1CC surface retained long, linear grooves associated with hot rolling and plastic deformation, with no obvious loosely adhered contaminants after cleaning. Localized bright features were also resolved. Because the BE yield decreases with decreasing mean atomic number, an oxide-enriched region would not be expected to produce positive BE contrast. Accordingly, these features are interpreted as contrast effects arising from local charging and edge-related topography rather than as compositional evidence. *HF* etching (2AE) exposed grain boundaries and generated a characteristic microtexture by dissolving the surface oxide and reacting with titanium according to eq (1):

1 $$Ti+3HF\to Ti^{3+}+1.5H_{2}+3F^{-}$$

**Figure 2:**
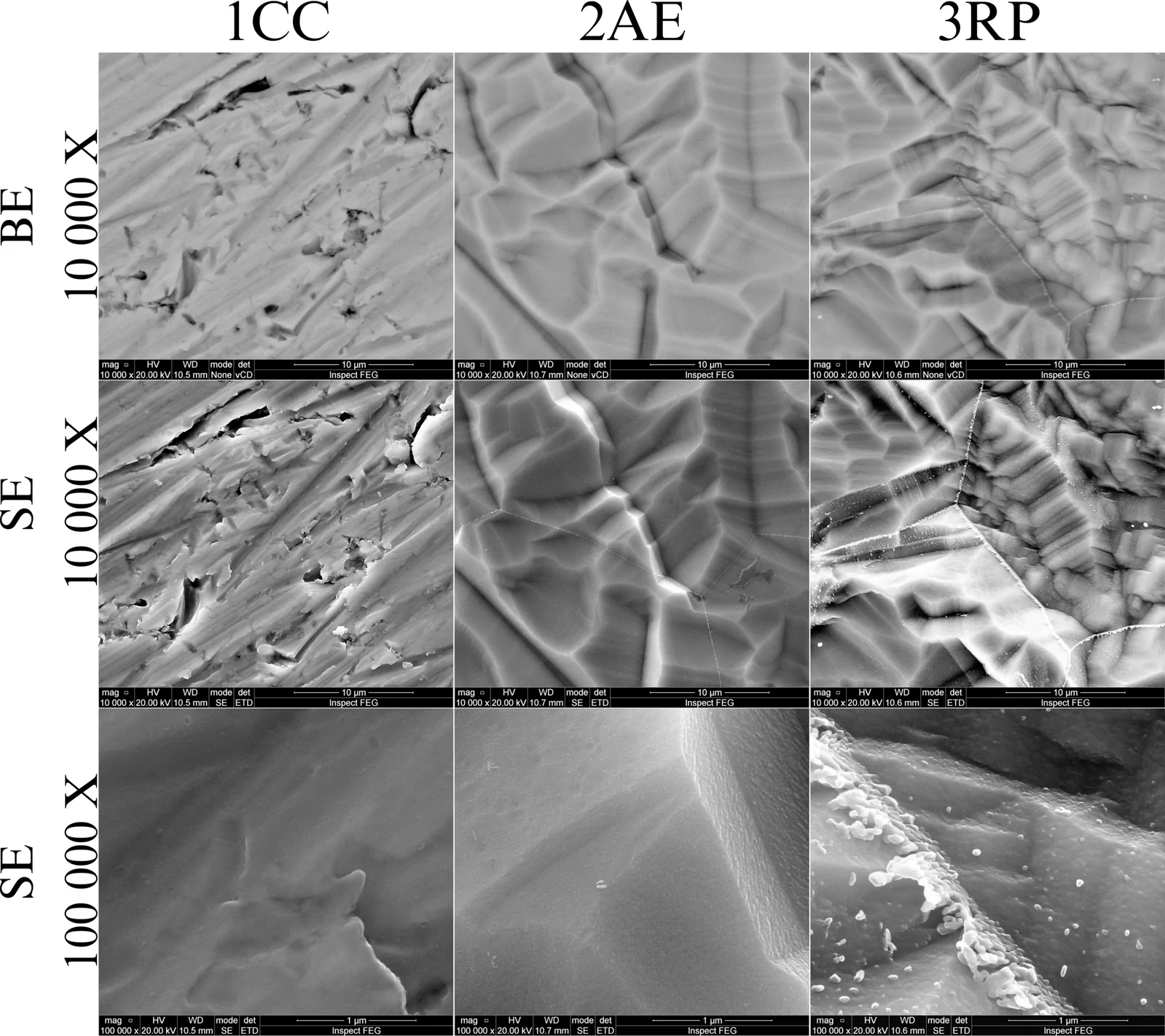
Surface morphology of titanium disks after different surface treatments. FESEM micrographs of 1CC, 2AE, and 3RP acquired in BE and SE modes at 20 kV, at 10,000× (scale bars 10 μm) and 100,000× SE (scale bars 1 μm). The surface 1CC shows rolling-induced grooves and localized bright features. The 2AE shows band-like corrosion grooves formed by preferential dissolution of the *α-Ti* matrix along grain boundaries. 3RP preserved this morphology while showing irregular bright features, because the BE yield decreases with decreasing mean atomic number. These are reported as contrast effects arising from local charging and edge-related topography rather than as compositional evidence. Surface chemistry is established by XPS (Figures 8 and 9).

In the present work, *HF* preferentially attacked the *α-Ti* matrix and propagated along grain boundaries, producing band-like corrosion grooves, consistent with the preferential dissolution of the α phase reported for *HF*-treated titanium. The resulting *Ti* ^*3+*^ species can subsequently oxidize in air and interact with *F* ^*–*^.^51^ This etched morphology was largely preserved after plasma activation. The 3RP surface remained similar to 2AE but exhibited irregular bright regions, whose chemical origin is established by XPS rather than by imaging contrast.

Laser-cutting artifacts are shown in Figure 3. Resolidified melt droplets and dross were present on the 1CC surface. *HF* etching exposed the internal nanostructure of these features and the underlying substrate, revealing a porous, honeycomb-like morphology within the dross and an acicular nanotexture on the titanium surface. Subsequent plasma activation preserved the overall nanostructure but blunted the acicular tips and increased the local brightness. These observations indicate limited topographical modification rather than a loss of the *HF*-generated morphology. Tip blunting was the only geometric change resolved after 3RP, and the associated chemical modification is addressed in the Surface Chemistry section.

**Figure 3:**
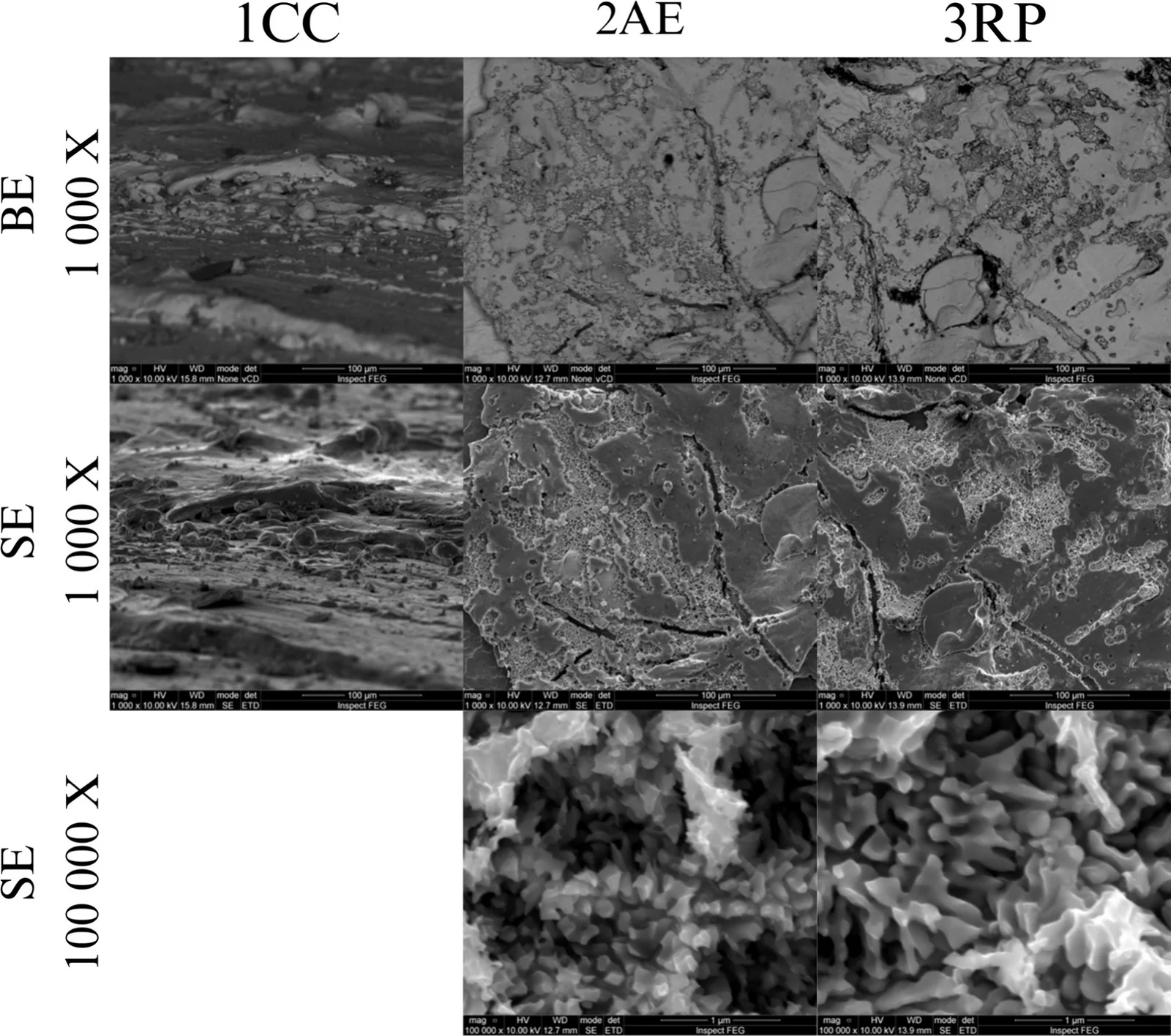
Nanostructural changes in laser-induced surface defects after chemical and plasma treatments. FESEM images of 1CC, 2AE, and 3RP in BE and SE at 10 kV, at 1,000× (scale bars 100 μm), and 100,000× (scale bars 1 μm). The tilted 1CC image (stage tilt ≈ 12°) highlights resolidified melt pools and dross-related defects generated during laser cutting. 2AE exposes a sharp acicular nanotexture on the titanium substrate and a porous, honeycomb-like structure within the dross droplets. 3RP blunts the acicular features and increases local brightness. As in Figure 2, the observed brightness is interpreted as contrast effect and is not considered evidence of oxidation.

#### Surface Roughness

Surface roughness was evaluated using the arithmetical mean deviation of the assessed profile (*Ra*), the root means square deviation of the assessed profile (*Rq*), and maximum height of the profile (*Rz*), as shown in Figure 4. All three parameters increased by approximately 86% from 1CC to 2AE (*Ra*, 0.40 ± 0.06 → 0.74 ± 0.03 μm; *Rq*, 0.52 ± 0.10 → 0.96 ± 0.06 μm; *Rz*, 2.77 ± 0.58 → 5.13 ± 0.64 μm), whereas subsequent plasma activation added no more than 9% (*Ra*, 0.81 ± 0.03 μm; *Rq*, 1.02 ± 0.05 μm; *Rz*, 5.43 ± 0.58 μm). Similar microtextured titanium surfaces have been associated with altered wettability and cellular adhesion; however, cellular responses were not evaluated in the present study.^10^

**Figure 4:**
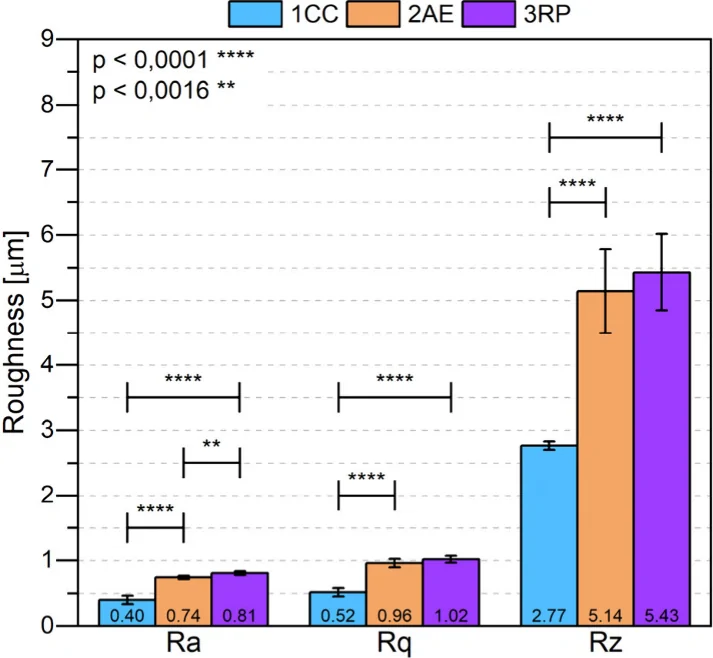
Surface roughness of titanium disks subjected to control cleaning (1CC), *HF* etching (2AE), and *HF* etching followed by plasma activation (3RP). Surface roughness parameters *R*_*a*_, *R*_*q*_, and *R*_*z*_ were measured by contact profilometry (λ_*c*_ = 0.8 mm, λ_*s*_ = 2.5 μm, an evaluation length of three sampling lengths). *Kruskal–Wallis* test, *p* < 0.0001, followed by *Dunn’s* post hoc test; all pairwise differences were significant for *R*_*a*_, whereas 2AE and 3RP did not differ significantly for *R*_*q*_ or *R*_*z*_ (adjusted *p* = 0.0656 and 0.9161, respectively). Data is presented as mean ± SD; *n* = 60, 60, and 20 independent disks for *R*_*a*_, *R*_*q*_, and *R*_*z*_, respectively.

Because the *Ra*, *Rq*, and *Rz* data sets did not meet the normality assumptions of the *Anderson–Darling*, *D’Agostino–Pearson*, *Shapiro–Wilk*, and *Kolmogorov–Smirnov* tests (*p* < 0.05), the groups were compared using the *Kruskal–Wallis* test (*k* = 3; *N* = 140), followed by Dunn’s post hoc test. A significant overall difference was detected for *Ra* (*H* = 114.3; *p* < 0.0001; *ε* ^2^ = 0.82), with significant differences among all pairwise comparisons (adjusted *p* ≤ 0.0016). Significant overall differences were also detected for *Rq* (*H* = 107.5; *ε* ^2^ = 0.77), and *Rz* (*H* = 102.0; *ε* ^2^ = 0.73; both *p* < 0.0001), 2AE and 3RP differed from 1CC (adjusted *p* < 0.0001), whereas no significant difference was detected between 2AE and 3RP (adjusted *p* = 0.0656 and 0.9161, respectively).

*HF* etching therefore produced the principal increase in surface-height variation, whereas plasma activation caused a smaller, parameter-dependent modification of the etched microtopography. This parameter dependence is consistent with the measurement bandwidth: with λ_*s*_ = 2.5 μm and a finite stylus tip radius, contact profilometry cannot resolve the nanoscale features imaged by FESEM at 100,000× magnification. Accordingly, the tip blunting observed after plasma activation would not be expected to contribute measurably to *Rq* or *Rz*. The limited topographic effect of plasma exposure is consistent with reports that nonthermal plasma activation preserves the pre-existing titanium topography while modifying only the outermost surface layer.^30,31,52^

#### Surface Hydrophilicity

Surface wettability progressively increased after *HF* etching and plasma activation, as shown in Figure 5A. The 1CC group exhibited a contact angle of θ = 47.1° ± 4.3°, corresponding to intermediate wettability with a tendency toward hydrophilicity and below the 63° reported for isopropanol-cleaned pure titanium, consistent with the additional detergent and ultrasonic deionized-water steps applied here.^14^ *HF* etching reduced the contact angle to θ = 23.2° ± 6.6° for 2AE, attributed partly to the generated surface microtexture.^10,53^ Plasma activation of the 3RP group produced a superhydrophilic surface, with θ = 13.5° ± 1.0° (90% disks at θ ≤ 15°, corresponding to the upper bound of the ″excellent wetting″ criterion [10–20°] in **ASTM D7334**, which was adopted here as the threshold for superhydrophilicity in engineered surfaces. Plasma activation also refined the wetting distribution: the coefficient of variation decreased from 28.5% for 2AE to 7.2% for 3RP, while the total range narrowed from 36.5° to 3.7°. The proportion of disks meeting the superhydrophilic criterion increased from 5% (2AE) to 90% (3RP). This behavior is consistent with the combined effects of micro/nanoscale topography and plasma-induced changes in oxide chemistry and surface polarity.^54^

**Figure 5:**
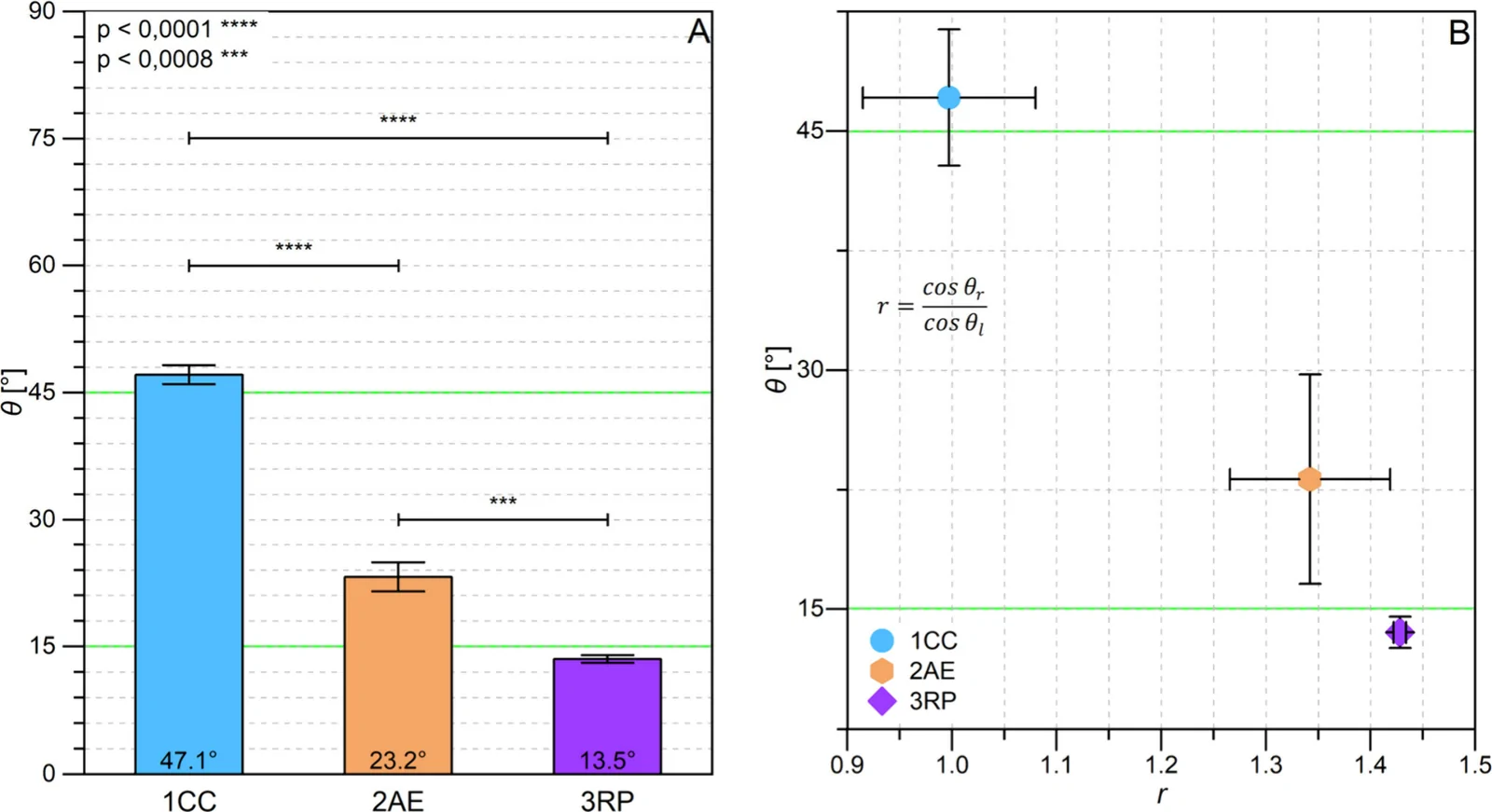
Surface characterization of titanium disks subjected to 1CC, 2AE, and 3RP: (A) Contact angle (θ). *Kruskal–Wallis* test, *χ* ^*2*^ = 111.8, *p* < 0.0001, followed by *Dunn’s* test, adjusted *p* ≤ 0.0008, all pairwise differences significant. (B) Relative wetting ratio (*r*), calculated with the *Wenzel* expression using the 1CC group mean as reference. Because *r* is an algebraic transform of the values in panel A, it carries the same rank information; therefore, no separate statistical test is reported. Data are represented as mean ± SD; *n* = 60, 60, and 20 independent disks for 1CC, 2AE, and 3RP, respectively.

Because the contact-angle data sets showed heterogeneous distributions and did not consistently meet normality assumptions, the groups were compared using the *Kruskal–Wallis* test (*k* = 3; *N* = 140). A significant overall difference was detected (*χ* ^*2*^ = 111.8; *p* < 0.0001). *Dunn’s* post hoc test identified significant differences between 1CC and 2AE (adjusted *p* < 0.0001), 1CC and 3RP (*p* < 0.0001), and 2AE and 3RP (p = 0.0008).

Relative wetting (*r*) was calculated as r = cosθ_*r*_/cosθ_*Y*_, where θ_*r*_ is the apparent contact angle on the rough surface and θ_*Y*_ is the intrinsic contact angle of the smooth reference surface.^55^ The 1CC condition — despite retaining measurable residual roughness (*Ra* = 0.40 ± 0.06 μm) and laser-processing-related surface features (Figures 2 and 3) — was adopted as the operational reference. Because θ_*Y*_ was fixed at the 1CC group mean, *r* is an exact algebraic transform of θ rather than an independent measurement of the actual-to-geometric area ratio defined by *Wenzel*:^55^ Consequently, its rank-based statistics are identical to those of θ (*χ* ^*2*^ = 111.8; *p* < 0.0001) and are not reported separately. As shown in Figure 5B, the derived values are *r* = 1.00 ± 0.08 for 1CC to 1.34 ± 0.08 for 2AE and 1.43 ± 0.01 for 3RP. Whether this increase reflects a purely topographic amplification is examined in the Discussion.

The long-term recovery of surface hydrophilicity was evaluated over eight plasma-activation cycles, comprising the initial activation and seven reactivations applied after ambient storage intervals of 1, 2, 7, 14, 21, 28, 35, and 42 days, corresponding to cumulative storage times of 1, 3, 10, 24, 45, 73, 108, and 150 days (Figure 6A). Immediately after each activation or reactivation, all 20 disks exhibited hydrophilic or superhydrophilic behavior, and the mean contact angles remained between 12.16° ± 0.70° and 13.54° ± 0.98° across the first seven cycles before rising to 16.19° ± 2.07° at the final cycle. In the 21-day-interval cycle, 40% of 20 droplets spread completely and were recorded at the lower measurement limit of the instrument (θ < 10°) in 30 s. The mean of that cycle (9.23° ± 8.60°) is therefore left-censored, and its median (12.48°) is consistent with the adjacent cycles. Nevertheless, a significant treatment-time effect was detected by the *Kruskal–Wallis* test (*H* = 55.21; *p* < 0.0001). *Dunn’s* post hoc test showed that the contact angle measured after the final reactivation was significantly higher than those measured after all preceding cycles (*p* < 0.05). The proportion of disks meeting the superhydrophilic criterion (θ ≤ 15°) was 85% of 20 in the first seven cycles but fell to 25% of 20 in the final cycle, indicating that reactivation efficacy was fully preserved through 108 days of cumulative storage and declined thereafter.

**Figure 6:**
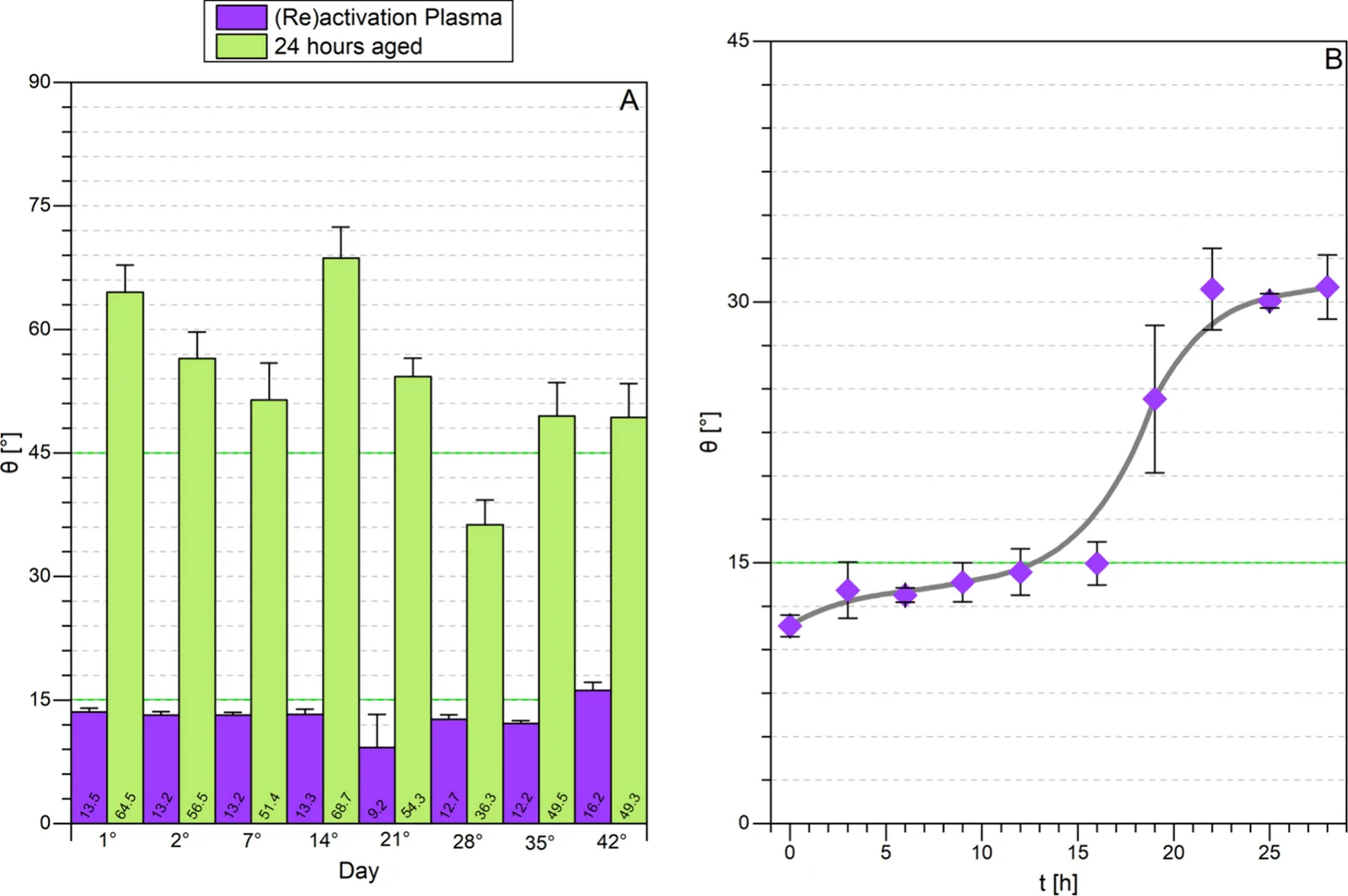
Hydrophilicity of plasma-activated titanium disks (3RP): (A) Recovery after sequential reactivations applied at ambient storage intervals of 1, 2, 7, 14, 21, 28, 35, and 42 days (cumulative storage: 1, 3, 10, 24, 45, 73, 108, and 150 days), measured immediately after each reactivation and after 24 h of ambient exposure. Droplets that spread completely were recorded at the lower instrument limit; this occurred for 40% of droplets in the 21-day-interval cycle (*Kruskal–Wallis*, *p* < 0.0001; the final reactivation differed from all preceding cycles, *Dunn’s* *p* < 0.05). (B) Short-term decay in ambient air without reactivation, measured repeatedly on the same disks, indicating a stability window of approximately 16 h. Mean ± SD; *n* = 20 disks in (A) and *n* = 3 in (B).

After 24 h of ambient exposure, the surfaces returned to the intermediate band (45° < θ ≤ 90°) in all cycles except the 28-day-intercal cycle, which remained hydrophilic (36.29° ± 6.40°). Significant differences among the temporal conditions were detected by the *Kruskal–Wallis* test (*H* = 92.95; *p* < 0.0001), and *Dunn’s* post hoc test (*p* < 0.05). The 24-h contact angle did not increase across the series and, if anything, decreased with cumulative storage time (*Spearman ρ* = −0.74; *p* = 0.037). These results indicate that plasma reactivation repeatedly restored surface hydrophilicity, without evidence of a cumulative loss of 24-h retention over the 150-day period.

The loss of hydrophilicity observed within 24 h is consistent with the progressive absorption of atmospheric contaminants, particularly hydrocarbons, which mask or modify the high-energy polar sites generated on the activated *TiO*_2_-rich surface and restrict the water spreading.^16,43^ This readsorption-driven decay parallels reports in which plasma- or ozone-generated oxidizing species transiently increase surface polarity by removing adsorbed hydrocarbons and modifying the oxide layer, an effect that is progressively lost as the surface reverts toward its pretreatment chemical and electrostatic stage upon environmental exposure.^20,56,57^ Although surface hydroxylation may contribute to the initial wettability, the regeneration of hydroxyl groups during successive plasma-reactivation cycles would indicate physicochemical reactivation rather than complete or permanent recovery of the initial surface state.

Reported stability windows for plasma- or UV-activated titanium and related oxides range from persistence checks limited to approximately 1h,^32^ through 24-h-to-7-day evaluations of single and combined surface treatments,^58^ to 21 days of wet storage, during which the contact-angle variation was confined to 7–12°,^44^ multiweek stability that was strongly dependent on the crystalline phase of the surface oxide,^59^ and 30 days of air storage, during which the polar surface energy was largely lost within the first 5–7 days.^60^ In clinical practice, this decay has motivated chairside plasma application immediately before implant placement, a protocol associated with progressive increases in the implant stability quotient over an 8-week healing period in a retrospective cohort.^61^ Because every reactivation interval applied here from the third cycle onward exceeded that 5–7 day recovery window, each reactivation acted on a surface that had already undergone essentially complete hydrophobic recovery. The restoration of θ to 12–13° across seven consecutive cycles therefore reflects repeatable reactivation from a fully aged baseline rather than the persistence of a single activation.

Wet storage may provide greater long-term stability because liquid storage after plasma activation can limit atmospheric hydrocarbon readsorption on *TiO*_2_-based titanium surfaces.^44^ This storage-dependent stabilization is consistent with reports showing that wet or vacuum storage of UV- or plasma-activated titanium and zirconia surfaces preserved hydrophilicity for several weeks, whereas dry ambient storage under otherwise comparable conditions resulted in progressive hydrophobic recovery.^62,63^ Accordingly, the dry aging conditions employed here represent a conservative assessment of plasma-induced hydrophilicity under ambient exposure.

Short-term wettability measurements on three disks aged without reactivation further defined the postactivation stability window (Figure 6B). The mean contact angle increased from 11.37° ± 0.62°, immediately after activation, to 14.96° ± 1.25° at 16 h, remaining at or below the superhydrophilic threshold. It then increased sharply to 24.42° ± 4.24° at 19 h and 30.75° ± 2.35° at 22 h, after which it plateaued at 30.07° ± 0.41° at 25 h and 30.86° ± 1.85° at 28 h. This evolution is consistent with progressive physicochemical re-equilibration of the activated surface through atmospheric readsorption,^16,43^ and the plateau indicate that readsorption reaches saturation before the surface reverts to the wettability of the untreated control. A similar hydrocarbon-driven, reorientation-related aging process has been described across other plasma-activated titanium surfaces, including substrates evaluated under controlled-temperature reactive plasma exposure,^60^ machined and rough titanium discs subjected to vacuum and vortex plasma discharges,^33,52^ nonthermal atmospheric plasma-treated implant surfaces compared with commercial controls,^64^ low-temperature plasma-modified dental implant surfaces more broadly,^65^ and nonthermal plasma-processed functional materials in general, for which a three-stage absorption–reaction–degradation model of surface aging has been proposed.^37^ Thus, plasma activation provided a practical window of approximately 16 h for subsequent surface use or experimental functionalization, although the biological relevance of this temporal window was not evaluated.

#### Surface Crystallinity

The raw XRD and GIXRD patterns obtained from the sequentially treated titanium disk are presented in Figure 7. In both configurations, the patterns were dominated by reflections from hexagonal close-packed *α-Ti* at approximately 35.1° (100), 38.4° (002), and 40.2° (101), in agreement with previous reports.^14,44,46,54^ Treatment-dependent changes were observed in the relative prominence of these reflections after *HF* etching and plasma activation. Because no quantitative crystallographic texture analysis was performed, these variations are described as a redistribution of diffraction intensities rather than definitive evidence of a newly developed preferred orientation. Acid-etching protocols employing hydrofluoric or other strong acids have been reported to generate secondary titanium-hydride reflections in some acid-etched titanium systems, a possibility that was not specifically investigated here and that represents a further limitation in interpreting treatment-dependent intensity changes in the 2AE condition.^63^ Because such a hydride layer forms during the etching step itself and is confined to a thin subsurface region,^63^ any contribution of this type would be superimposed on the *α-Ti* reflections in the present raw patterns and could not be resolved without phase-fraction refinement. The intensity redistribution observed in 2AE is therefore treated as an unresolved combination of crystallographic texture and possible hydride contributions.

**Figure 7:**
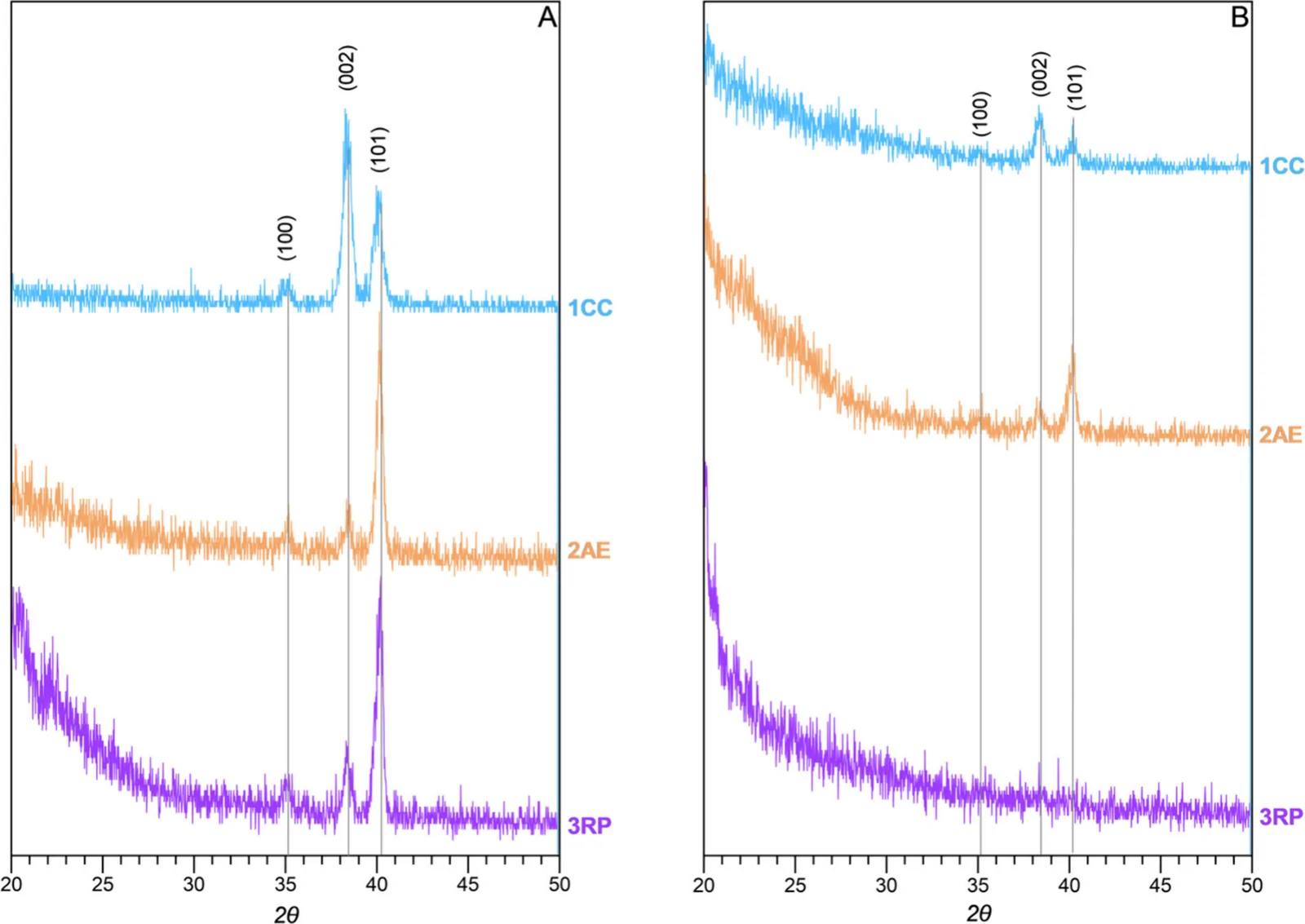
Raw XRD and GIXRD patterns of the sequentially treated titanium disk under the 1CC, 2AE, and 3RP conditions. (A) Conventional XRD and (B) GIXRD acquired at a fixed incidence angle of 1°. Reflections at approximately 35.1°, 38.4°, and 40.2° are assigned to the (100), (002), and (101) planes of *α-Ti*. The increased intensity in the low-angle region of the 3RP patterns is an elevated diffuse scattering/background contribution, not a discrete reflection. No reflections attributable to crystalline anatase or rutile *TiO*_2_ were resolved within the 20–50° range examined. Patterns are vertically offset for clarity.

No resolved reflections attributable to crystalline anatase or rutile *TiO*_2_ were detected within the investigated *2θ* range. The 20–50° window examined here encompasses the reflections near 25° and 28° that have been used to identify anatase and rutile on titanium implant surfaces,^62^ so the absence of such reflections is informative within the detection limits of the measurement rather than being a consequence of the selected range. The 3RP pattern exhibited an elevated and continuously decreasing intensity contribution between approximately 20 and 25°, which was more evident under grazing-incidence geometry because of the greater relative sensitivity to the near-surface region obtained at a 1° incidence angle. However, geometry-dependent background and surface-related diffuse scattering may also contribute to this response. Because only raw patterns were evaluated, without background subtraction or integration of the low-angle region, this contribution cannot be quantitatively separated from the underlying background and is not considered definitive evidence of amorphous *TiO*_2_.

Even under grazing incidence, however, the diffracting volume remains dominated by the metallic substrate: reducing the incidence angle to 1° shortens the probed depth by roughly an order of magnitude relative to the symmetric Bragg–Brentano configuration, yet the resulting depth remains far greater than the thickness of a plasma-grown surface oxide. An oxide layer of a few nanometres thick therefore represents a negligible fraction of the diffracting volume, below the phase fraction ordinarily required for detection by laboratory diffractometry. The absence of anatase or rutile reflections places an upper bound on the amount of crystalline oxide present rather than excluding crystalline order within the outermost layer.

This absence of a resolved crystalline-oxide signal parallels reports in which XRD analysis of comparably *HF*- or plasma-treated titanium surfaces detected no peaks attributable to anatase or rutile *TiO*_2_, with the diffraction pattern remaining dominated by the metallic substrate,^62^ as well as reports in which cold atmospheric plasma treatment of titanium disks produced no discernible alteration in crystalline structure relative to untreated controls.^29^ In contrast, crystalline *TiO*_2_ reflections have been reported for titanium surfaces subjected to plasma treatment under substrate heating (approximately 100–200 °C), with grazing-incidence XRD and Raman spectroscopy showing a temperature-dependent increase in tetragonal *TiO*_2_ content,^60^ and for UV- or plasma-treated sputtered anatase and rutile thin films, in which the pre-existing crystalline phase itself governed long-term hydrophilic stability.^59^ Because the present protocol involved a short, ambient-temperature plasma exposure of the native oxide rather than treatment of a heated substrate or a predeposited crystalline film, these temperature- or phase-dependent effects are not directly transferable to the 3RP condition evaluated here. The comparison is nonetheless informative in the opposite direction: in those systems, crystalline-phase development was the factor governing the persistence of hydrophilicity, whereas the surfaces studied here sustained repeated recovery of the superhydrophilic state across eight activation cycles without any detectable crystalline oxide. Reactivation in the present system therefore appears to operate through changes in the chemical state of the outermost layer rather than through the formation or growth of a crystalline oxide phase.

XPS independently indicated a *TiO*_2_-rich plasma-activated surface but did not establish long-range crystallographic order. The two techniques probe different depth regimes of the same surface — photoemission reports the chemical state of the outermost few nanometres, whereas both diffraction geometries integrate over a volume dominated by *α-Ti* — so their divergence is an expected consequence of the sampling depths rather than an inconsistency between the data sets. The combined results therefore support the formation of an oxide-rich surface after plasma activation, while the structural order of this oxide cannot be determined from the present diffraction data set.

#### Surface Chemistry

The surface chemical evolution of the sequentially treated titanium disk was evaluated by XPS under 1CC, 2AE, 3RP, and 3RP-A conditions. The survey spectra in Figure 8A showed *C 1s*, *O 1s*, and *Ti 2p* signals under all conditions, consistent with adventitious carbon, surface oxygen species, and the titanium substrate. Treatment-dependent signals included *N 1s* at approximately 400 eV for 1CC and 3RP-A and *F 1s* at approximately 685 eV for 2AE. The corresponding binding energies, fwhm values, and relative peak-area contributions are summarized in Table S1 of the Supporting Information.

**Figure 8:**
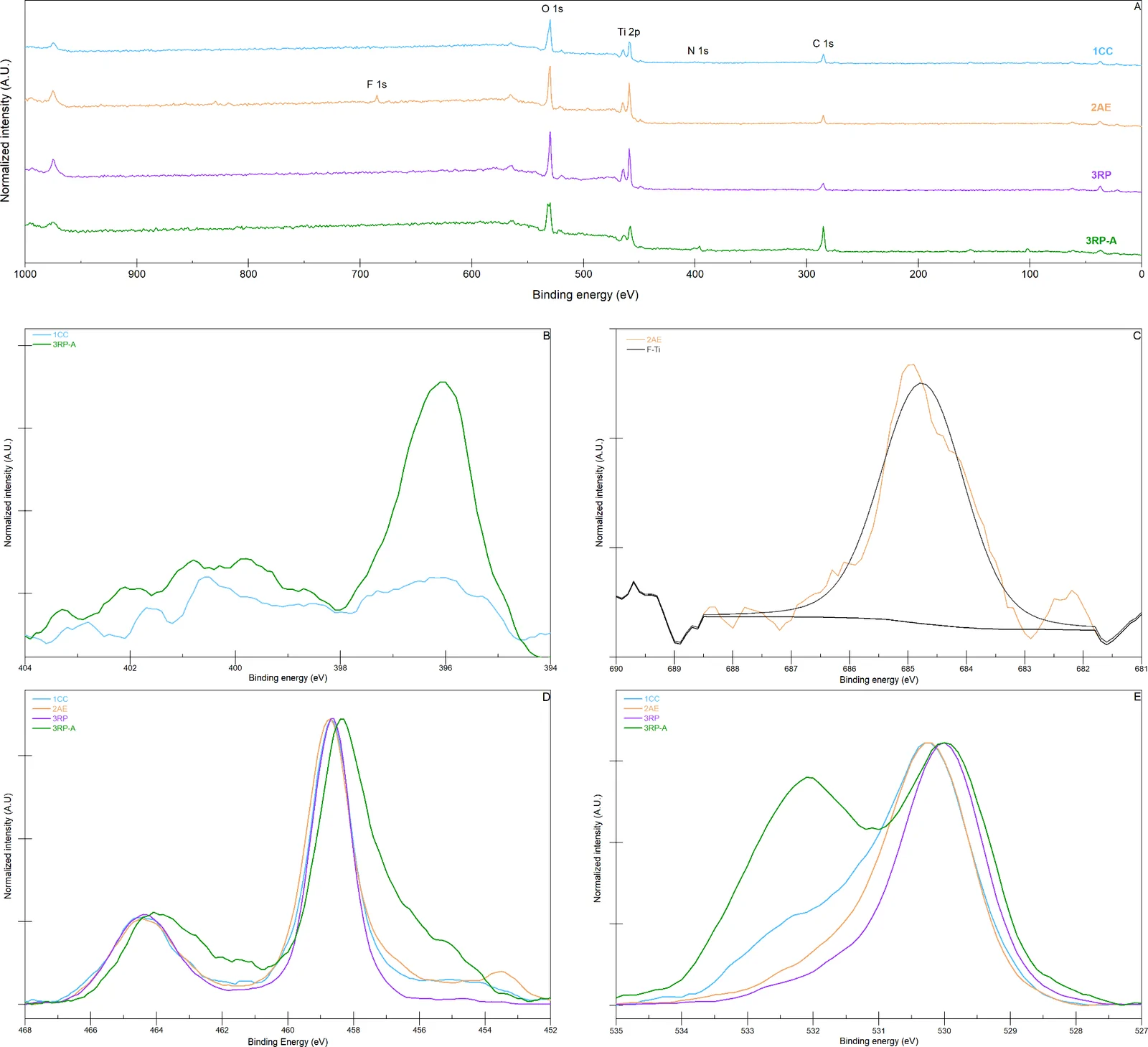
X-ray photoelectron spectroscopy (XPS) analysis of titanium surfaces after different treatments. (A) Survey spectra showing *Ti 2p*, *O 1s*, adventitious *C 1s*, and treatment-dependent *N 1s* and *F 1s* signals. (B) *N 1s* spectra of 1CC and 3RP-A; no *N 1s* signal was resolved for 2AE or 3RP. (C) *F 1s* spectrum for 2AE, evidencing *Ti–F* species; no *F 1s* signal was resolved for 3RP. (D) *Ti 2p* envelopes dominated by *TiO*_2_, with treatment-dependent oxynitride, fluoride, and metallic *Ti* states. (E) *O 1s* envelopes showing treatment-dependent changes, comprising lattice *Ti–O*, an unresolved *O–N/O–H* contribution, and adsorbed water or other oxygen-containing species. All spectra were acquired from a single sequentially treated disk (*n* = 1) after approximately 48 h of vacuum residence. The 3RP-A condition included post-3RP cleaning and controlled storage and is therefore not a pure 24 h atmospheric-aging condition.

The *C 1s* region showed no marked treatment-dependent differences and was attributed predominantly to adventitious carbon. This result reflects the acquisition protocol rather than necessarily the activated state itself: every spectrum was recorded after atmospheric transfer and approximately 48 h of vacuum residence, well beyond the 16 h stability window established in Figure 6B. The *C 1s* signal therefore describes a re-equilibrated surface rather than the freshly activated condition corresponding to the contact-angle measurements and should not be interpreted as evidence against hydrocarbon removal during the discharge. Studies in which surface analysis was performed promptly after treatment report the opposite behavior: a 19.7% relative reduction in surface carbon after plasma treatment in an air-fed discharge,^32^ a significant decrease in the hydrocarbon component of the *C 1s* spectrum accompanied by a increase in the *O–Ti* contribution after vacuum plasma treatment,^33^ and a 28.1% reduction in carbon content measured by energy-dispersive X-ray spectroscopy.^52^ The absolute carbon levels reported in these studies differ by more than a factor of 2 and were obtained by techniques with different sampling depths; they are therefore interpreted here as evidence of a consistent direction of change rather than as values directly comparable with the present data set.

High-resolution *N 1s* spectra of 1CC and 3RP-A (Figures 8B) contained contributions assigned to *N–Ti* and *N–O* bonding environments at approximately 396.2 and 400.1 eV, respectively.^66–69^ No *N 1s* signal was resolved for 2AE or 3RP. The absence of nitrogen after plasma activation is noteworthy because the discharge was generated in air, in which reactive nitrogen species were necessarily present in the plasma volume. Their nonincorporation into the surface is consistent with a mechanism in which excited nitrogen species react with adsorbed hydrocarbons and leave the surface as volatile products, a route proposed for air-fed plasma treatment of titanium implants.^32^ The discharge applied here therefore appears to have acted predominantly as an oxidative cleaning step rather than a nitriding process, which is also consistent with the *Ti 2p* envelope of 3RP being dominated by *TiO*_2_-related contributions. The reappearance of nitrogen-related contributions in 3RP-A is attributed to the additional cleaning, desiccator-storage, and vacuum-equilibration sequence applied to that condition rather than to the plasma treatment itself.

The 2AE condition exhibited an *F 1s* peak at 684.8 eV, consistent with *Ti–F* species formed during *HF* etching.^13,70–73^ No resolved *F 1s* signal was detected for 3RP in either the survey spectrum or the dedicated high-resolution scan, indicating that any residual fluorine was below the practical XPS detection limit under the applied acquisition conditions; this finding does not demonstrate complete fluorine removal (Figures 8C).

*Ti 2p* and *O 1s* core-level regions. The comparative *Ti 2p* and *O 1s* spectral envelopes are presented in Figures 8D and 8E, and corresponding curve-fitting are shown in Figures 9A and 9B.

**Figure 9:**
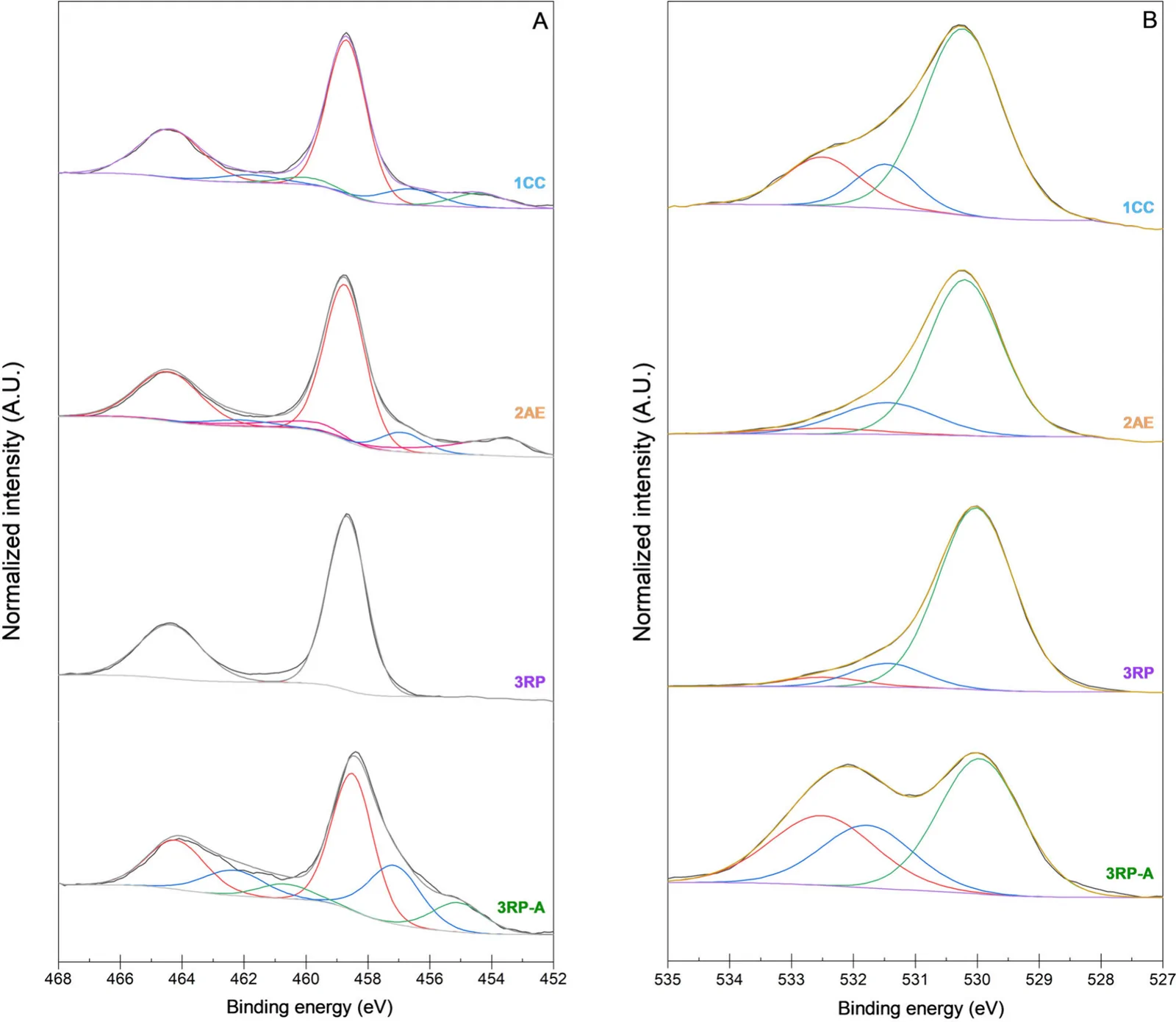
High-resolution XPS spectra and curve-fitting results for the sequentially treated titanium surface (*n* = 1, as in Figure 8). (A) *Ti 2p* and (B) *O 1s* spectra of 1CC, 2AE, 3RP, and 3RP-A. Experimental spectra, total fitted envelopes, and backgrounds are shown in black, orange, and gray, respectively, and the spectra are normalized and vertically offset for visual comparison. Binding energies, fwhm values, and relative fitted areas are summarized in Table S1.

As shown in Figures 8D and 9A, in the *Ti 2p* region, all conditions exhibited *TiO*_2_-related signals at approximately 458.7 and 464.4 eV.^13,66,68^ ^,^ ^74,75^ The 1CC spectrum also contained components assigned to *TiON* and *TiNO*, with doublets at 456.6/461.8 and 454.4/460.0 eV, respectively.^66–68,74–76^ Together with the *N 1s* results, these components suggest nitrogen-containing contamination associated with the laser-cutting process, which may become trapped within the porous, honeycomb-like dross nanostructure exposed by *HF* etching in Figure 3. For 2AE, a *Ti–F*-related doublet was detected at approximately 456.9 and 462.1 eV.^13,70–72^ Metallic *Ti* ^0^-related components were also observed at 453.5 and 459.6 eV, accounting for 18.6% of the fitted *Ti 2p* area and consistent with thinning or partial removal of the surface oxide during *HF* etching.^13,70,71^ The *Ti* ^0^ contribution was detected only for 2AE and was interpreted together with the *Ti–F*-related doublet and the resolved *F 1s* signal as evidence of *HF*-induced modification of the near-surface oxide layer.

The fitted *Ti 2p* components presented in Figure 9A show that no *Ti* ^0^ component was retained in the final fitting model for 3RP, whose *Ti 2p* envelope was accounted for entirely by *Ti* ^*4+*^/*TiO*_2_-related contributions (100% of the fitted area; Table S1), compared with 78.6% for 1CC and 71.5% for 2AE. The recovery of a fully oxidized *Ti 2p* envelope after a 1 min discharge, on a surface from which *HF* etching had exposed metallic titanium, is the most direct chemical evidence of the oxide-rich state referred to throughout this work. Alternative models containing *Ti* ^*3+*^ and/or *Ti* ^0^ were tested; although they reduced the residual of the fit, they converged to nonrobust minor contributions and physically inconsistent fwhm values and were therefore not retained, without being statistically excluded. A *Ti* ^*4+*^-to-*Ti* ^*3+*^ reduction accompanied by oxygen-vacancy formation has been proposed as a wettability-relevant mechanism in other plasma-activated titanium systems, including vacuum-assisted plasma treatment of implant surfaces^32^ and in-pack cold atmospheric plasma treatment of titanium disks, in which a measurable *Ti* ^*3+*^ fraction and a concurrent increase in *O–H*-related contributions were resolved.^29^ Three considerations are relevant to the absence of such a component here. First, the region between 456 and 457 eV in which reduced titanium states have been reported also accommodates the *TiON* and *Ti–F* doublets fitted for 1CC and 2AE at 456.6 and 456.9 eV, respectively; assignment within this window is therefore not unique and depends on the complementary *N 1s* and *F 1s* evidence. Second, *Ti* ^*3+*^ states and their associated oxygen vacancies are metastable and can undergo reoxidation upon exposure to atmospheric oxygen,^51^ whereas every spectrum reported here was acquired after atmospheric transfer and approximately 48 h of vacuum residence. Third, the reported *Ti* ^*3+*^ fractions are strongly model-dependent: values of 36.4% have been reported for untreated titanium controls and 43.9% after cold atmospheric plasma treatment,^29^ making these values not directly comparable with the fitting convention adopted here. A reduced-titanium contribution is therefore neither adopted as part of the proposed mechanism nor excluded by the present data set. The 3RP-A spectrum contained *TiO*_2_-, *TiON*-, and *TiNO*-related components at similar binding energies, with the *TiO*_2_ contribution falling to 55.1% and nitrogen-related components accounting for the remaining 45.0%. Because this condition involved an additional cleaning, desiccator-storage, and vacuum-equilibration sequence, differences between 3RP and 3RP-A cannot be attributed exclusively to atmospheric aging. A shift of approximately – 0.18 eV in the *TiO*_2_-related *Ti 2p*_*3/2*_ and *Ti 2p*_*1/2*_ peak maxima (458.68 → 458.50 eV and 464.38 → 464.20 eV; Table S1) was also observed and was consistent with the increased nitrogen-related contribution at the surface.

The comparative *O 1s* envelopes and corresponding fitted components are shown in Figures 8E and 9B, respectively. The spectra were fitted using three components. The contribution at approximately 530.2 eV was assigned to lattice *Ti–O* bonds and remained the predominant component under all conditions, increasing from 69.9% for 1CC to 75.6% for 2AE and 84.7% for 3RP (Table S1).^16,66,67,75^ The component near 531.5 eV falls within overlapping binding-energy ranges reported for *O–N* and *O–H* species and was therefore represented as a single unresolved *O–N/O–H* contribution.^16,66,74,75^ A more granular three-component resolution of the O 1s envelope — comprising lattice *O* ^2–^, acidic bridging *OH*(s), and *Ti–OH* contributions — has been achieved for comparable *TiO*_2_-rich, plasma-activated titanium surfaces under conditions in which the *N 1s* contribution was negligible.^60^ That resolution was not attempted here because the treatment-dependent *N 1s* contributions detected in the 1CC and 3RP-A conditions preclude unambiguous separation of the *O–N* and *O–H* components within the present data set. For 1CC and 3RP-A, the *N 1s* signals and the *TiON*- and *TiNO*-related *Ti 2p* components support an *O–N* contribution within this envelope. For 2AE and 3RP, the absence of a resolved *N 1s* signal makes an *O–H* contribution chemically plausible, although this remains an indirect interpretation rather than a spectrally resolved assignment. For those two conditions, the fitted position of this component (531.46 and 531.45 eV) coincides with the 531.24–531.45 eV range reported for acidic bridging hydroxyl groups on plasma-treated titanium, and the decrease of this component from 2AE to 3RP together with the concurrent rise of the lattice *Ti–O* contribution follows the same direction as the enrichment in lattice O^2–^ and the reduction of acidic sites described for those surfaces.^60^ This correspondence is descriptive: the component remains an unresolved envelope and was not used as a quantitative measure of hydroxyl density. The component at approximately 532.5 eV was assigned to adsorbed water and oxygen-containing organic species.^66,70,74,75^ Its contribution decreased from 17.4% for 1CC to 4.3% for 2AE during acid etching and remained at a similar level after plasma activation (4.7% for 3RP); together with the predominance of *Ti–O*, this finding was consistent with a cleaner, *TiO*_2_-rich surface. A comparable reduction in hydrocarbon-related carbon species, accompanied by concurrent increases in *O–Ti* and hydroxyl contributions after plasma treatment, has been reported using an analogous *C 1s/O 1*s deconvolution scheme applied to titanium surfaces subjected to vortex and direct plasma discharge.^33^

Based on the fitted *O 1s* components in Figure 9B, a descriptive comparison was performed between the relative area of the unresolved *O–N/O–H* component and the mean contact angle for 1CC, 2AE, and 3RP. The corresponding peak-area contributions were 12.7%, 20.1%, and 10.7%, whereas the mean contact angles were 47.1° ± 4.3°, 23.2° ± 6.6°, and 13.5° ± 1.0°, respectively. The lowest contact angle was therefore observed for 3RP despite its lower 531.5 eV contribution relative to 2AE, indicating that no monotonic quantitative relationship was established for this component. Of the remaining two components, only the lattice *Ti–O* contribution followed the wettability sequence, increasing monotonically from 69.9% to 75.6% and 84.7% as the contact angle decreased across 1CC, 2AE, and 3RP; the adsorbed-species contribution decreased at the etching step, from 17.4% to 4.3%, and remained unchanged within the resolution of the fitting thereafter (4.7%). This concordance in direction is reported descriptively and was not subjected to inferential statistical testing. Because this component does not represent an exclusive *O–H* fraction, and because the XPS and wettability measurements were obtained from different experimental units and replication schemes, no inferential correlation was performed. Quantitative correlations between surface carbon content and contact angle have been established elsewhere using paired measurements obtained from the same specimens,^63^ an experimental design not adopted here because the sequential single-disk XPS protocol and the independent multidisk wettability data set followed different replication schemes. Superhydrophilicity cannot therefore be attributed to the 531.5 eV contribution alone and is interpreted as a multifactorial response involving the *TiO*_2_-rich activated surface, reduced adsorbed oxygen-containing species, changes in surface polarity, and micro/nanotopography, with hydroxylation remaining a chemically plausible contribution.^14,16,43^ ^,^ ^53,55^ The greater 532.5 eV contribution observed for 3RP-A (29.9%, against 4.7% for 3RP), accompanied by the fall of the lattice *Ti–O* contribution to 46.4%, is described as condition-dependent surface evolution rather than an effect attributable exclusively to atmospheric aging, although its direction matches the wettability decay documented in Figure 6B.

#### Mechanistic Interpretation of Plasma-Induced Surface Activation and Wettability Evolution

The proposed mechanism integrates the *HF*-generated microand nanotopography shown in Figures 2 and 3, the roughness data presented in Figure 4, the contact-angle results in Figure 5, and the XPS evidence presented in Figures 8D/E and 9A/B and Table S1. Titanium exposed to air is commonly described as a multilayer interface comprising a titanium oxide (*Ti*_*x*_ *O*_*y*_) layer covered by adsorbed water and hydrocarbons.^16,43^ *HF* etching modified this interface by removing or thinning the native oxide and generating a micro/nanotextured surface. The chemical signature of this step is unambiguous: the 2AE condition was the only one in which metallic *Ti* ^0^ was fitted (18.6% of the *Ti 2p* area), together with a *Ti–F* contribution of 9.9% and a resolved *F 1s* signal, while *Ra*, *Rq*, and *Rz* all increased by approximately 86%. The contact angle decreases from 47.1° to 23.2°.

During plasma activation, energetic ions, radicals, and other reactive species generated by the air-dominant glow discharge interacted with the near-surface oxide, removing adsorbed species and modifying the oxide chemistry and surface polarity.^43^ Three independent observations define the outcome of this step. First, the *Ti 2p* envelope of 3RP was accounted for entirely by *TiO*_2_-related components (100%), compared with 71.5% for 2AE, indicating that the metallic titanium and fluoride species exposed by etching were reoxidized within a 1 min exposure. Second, the lattice *Ti–O* contribution to the *O 1s* envelope increased to 84.7%, the highest value among the four conditions, while the component assigned to adsorbed water and oxygen-containing organic species remained low (4.7%). Third, no *N 1s* signal was resolved for 3RP even though the discharge was generated in air, indicating that the reactive nitrogen species present in the plasma consumed adsorbed hydrocarbons without being incorporated into the surface.^32^ The discharge therefore acted as a combined cleaning and oxidation step rather than as a deposition or nitriding process.

The decisive point is that this chemical transformation occurred without a corresponding topographical change. *Rq* and *Rz* did not differ significantly between 2AE and 3RP (Figure 4), so the roughness term was effectively constant across the two conditions, yet the contact angle decreased by a further 9.6°, corresponding to a 5.7% increase in cos θ. Roughness amplification alone therefore cannot account for the transition to superhydrophilicity, a conclusion further supported by the magnitude of roughening that would be required: reproducing the 3RP contact angle from the intrinsic value reported for flat *TiO*_2_ would require a roughness factor of approximately 2.9,^14^ far above the areal Wenzel factors at most 1.25 measured on *HF*-etched pure titanium with *Sa* between 0.7 and 4.7 μm.^10^ Because the *O–N* and *O–H* contributions near 531.5 eV were not spectrally resolved, the formation of a high density of terminal hydroxyl groups was not directly demonstrated, and surface hydroxylation is therefore considered a chemically plausible contribution rather than a quantitatively established mechanism. The wettability response is interpreted as the combined result of a *TiO*_2_-rich activated surface, reduced adsorbed oxygen-containing species, treatment-induced changes in surface polarity, and the *HF*-generated micro/nanotopography.^14,16,43^ ^,^ ^53,55^

During ambient exposure, progressive adsorption of atmospheric species can mask or modify high-energy polar sites, thereby increasing the contact angle and limiting the stability of the activated state, as shown by the short- and long-term wettability results in Figures 6A and 6B.^16,43^ The short-term series defines the kinetics of this process: the superhydrophilic state persisted for approximately 16 h, the contact angle then increased sharply between 16 and 19 h, and stabilized near 30° from 22 h onward, indicating that readsorption saturates well before the wettability of the cleaned control is recovered. The chemistry of the 3RP-A condition follows the same direction, with the lattice *Ti–O* contribution decreasing to 46.4% and the adsorbed-species contribution rising to 29.9%. Because that condition included an intervening cleaning and storage sequence, these values are not treated as isolated evidence of atmospheric aging; their direction, however, is consistent with the wettability data. The surface is therefore interpreted as a chemically dynamic interface in which plasma-induced cleaning and oxide activation govern the initial wettability response, whereas environmental adsorption progressively promotes hydrophobic recovery. Because each reactivation interval from the third cycle onward exceeded the reported 5–7-day window for hydrophobic recovery,^60^ every reactivation was applied on a fully aged surface, and the superhydrophilic state was nonetheless restored in seven consecutive cycles spanning 108 days of cumulative storage. The mechanism proposed here is therefore repeatable rather than persistent: it does not preserve the activated state, but it regenerates it.

From an application perspective, plasma-mediated physicochemical cleaning may be useful for reducing surface contamination before subsequent functionalization and biological testing. The scope and limitations of this interpretation are discussed in the following subsection.

#### Experimental Scope and Biological Limitations

The following limitations bound the interpretation of the present results. Surface morphology, roughness, and wettability were evaluated using multiple titanium disks, whereas the XRD/GIXRD and XPS data sets shown in Figures 7–9 were obtained from one sequentially treated disk. This within-specimen design reduced variability associated with the initial crystallographic texture and surface heterogeneity but did not provide independent replication for diffraction or surface-chemical measurements. Accordingly, these data sets were interpreted descriptively, without inferential statistical analysis. The short-term wettability series in Figure 6B was acquired from the same three disks at every time point and therefore constitutes repeated measures; those disks were dried under a stream of dry nitrogen after each measurement, a handling step not applied to the long-term reactivation series, so the two panels of Figure 6 are not directly comparable at equivalent exposure times. Because XPS and contact-angle measurements were obtained from different experimental units and replication schemes, no inferential correlation between surface chemistry and wettability was performed.

The interpretation is further bounded by the detection limits and the methodological constraints of the individual techniques. Contact profilometry is band-limited by the 2.5 μm short-wavelength cutoff and by the finite stylus tip radius and therefore cannot resolve the nanoscale features imaged by FESEM; the evaluation length also comprised three sampling lengths rather than the five specified as the default in **ISO 4288**. The raw XRD/GIXRD patterns were not subjected to background subtraction, quantitative texture analysis, or phase-fraction determination and therefore cannot establish the structural order of the plasma-induced oxide. The *2θ* range was additionally restricted to 20–50°, and, because the diffracting volume remains dominated by the metallic substrate even under grazing incidence, the absence of anatase and rutile reflections constrains the amount of crystalline oxide that can be present rather than excluding crystalline order within the outermost layer. All XPS spectra were acquired after atmospheric transfer and approximately 48 h of vacuum residence and therefore describe a re-equilibrated surface rather than the freshly activated state to which the contact-angle measurements correspond. The overlapping *O–N* and *O–H* contributions near 531.5 eV prevented direct quantification of surface hydroxylation, and the absence of a resolved *F 1s* signal for 3RP indicates that residual fluorine fell below the practical detection limit rather than that fluorine was removed completely. Finally, the relative wetting ratio in Figure 5B was calculated from the contact-angle data set using the 1CC group mean as the reference angle; it is therefore an algebraic transform of those data and not an independent measurement of the actual-to-geometric area ratio defined by the *Wenzel* model.

The 3RP-A condition also included an intervening cleaning, desiccator-storage, and vacuum-equilibration sequence and therefore should not be interpreted as an isolated atmospheric aging condition. In addition, protein adsorption, cellular responses, bacterial adhesion, microbiological sterility, and in vivo performance were not evaluated. Although previous studies have reported decontamination effects and improved biological responses for plasma-activated titanium surfaces,^26,27,36,43^ the present findings demonstrate only physicochemical surface cleaning, oxide activation, micro/nanotopographical modification, and enhanced wettability. Biological or clinical benefits should therefore not be inferred directly from the current data set and require dedicated biological validation.

## Conclusions

The hybrid treatment combined *HF*-induced microtexturing with reactive glow discharge plasma generated in a DC magnetron sputtering system operated as an air-fed, low-pressure source to modify the physicochemical properties of commercially pure grade 1 titanium. *HF* etching preferentially dissolved the α-Ti matrix along grain boundaries, producing band-like corrosion grooves and a hierarchical micro/nanotexture that accounted for the principal increase in surface roughness, with *R*_*a*_ increasing by 86%, from 0.40 ± 0.06 μm to 0.74 ± 0.03 μm. Plasma activation largely preserved this morphology while promoting localized oxidation and smaller further change in microtopography (*Ra* 0.81 ± 0.03 μm).

The mean water contact angle decreased from 47.1° ± 4.3° to 23.2° ± 6.6° and 13.5° ± 1.0°, yielding a superhydrophilic plasma-activated surface. Eight reactivation cycles spanning 150 days of cumulative ambient storage restored hydrophilicity, with efficacy preserved through 108 days and declining thereafter; the activated state was not permanent, persisting approximately 16 h.

Raw XRD and GIXRD patterns remained dominated by *α-Ti* and showed treatment-dependent changes in relative diffraction intensity, without resolved reflections attributable to crystalline anatase or rutile *TiO*_2_. The elevated low-angle contribution was interpreted as diffuse scattering rather than evidence of amorphous *TiO*_2_. XPS showed that *HF* etching generated *Ti–F*-related species and exposed a metallic *Ti* ^0^ contribution, neither of which was resolved after plasma activation, where the *Ti 2p* envelope was accounted for entirely by *Ti* ^*4+*^/*TiO*_2_-related contributions (100% of the fitted area). Adsorbed oxygen-containing species remained at 4.7%, against 4.3% for the etched and 17.4% for the cleaned control.

The superhydrophilic response is therefore attributed to the combined effects of *HF*-generated micro/nanotopography, oxide activation, reduced surface adsorption, and changes in surface polarity rather than to a single chemical component. These results establish DC magnetron-generated reactive glow-discharge plasma activation as a rapid physicochemical route for titanium surface engineering. Because biological responses were not evaluated, the findings should not be interpreted as evidence of an improved biological or clinical performance. Process scalability was not assessed, and the aged condition (3RP-A) included intervening cleaning, desiccator-storage, and vacuum-equilibration steps; therefore, differences between 3RP-A and 3RP cannot be attributed exclusively to ambient aging.

## Supplementary Material



## Notes

High-resolution XPS spectra of *Ti 2p*, *O 1s*, *N 1s*, and *F 1s* regions, combined XPS spectral panels, and XPS deconvolution parameters, including binding energies, fwhm values, and relative peak contributions (PDF)

## ○

Present address for Mickael Melo Linkiewicz: Materials Laboratory, Metallic Materials Engineering Nucleus, Polytechnic School, Pontifical Catholic University of Rio Grande do Sul (PUCRS), 90619-900, Porto Alegre, RS, Brazil

## ◆

Present address for Angelika von Schmude: Independent Researcher, Porto Alegre, RS, Brazil.

## ◑

Present address for Marcus Seferin: Chemometrics and Analytical Instrumentation Laboratory, Institute of Chemistry, Federal University of Rio Grande do Sul (UFRGS), 91501-970, Porto Alegre, RS, Brazil.

## ⬓

Present address for Roberto Hübler: Independent Researcher, Porto Alegre, RS, Brazil.

## Abbreviations

1CC: Control Cleaning
2AE: Acid Etching
3RP: Reactive Plasma Activation
3RP-A: Aged Reactive Plasma Condition
BE: Backscattered Electrons
DC-MS: DC Magnetron Sputtering
DI: Deionized
FESEM: Field-Emission Scanning Electron Microscopy
fwhm: Full Width at Half Maximum
GDP: Glow Discharge Plasma
GIXRD: Grazing Incidence X-ray Diffraction
HCP: Hexagonal Close Packed
HF: Hydrofluoric Acid
SE: Secondary Electrons
SLA: Sandblasted and Acid-Etched
UV: Ultraviolet
XPS: X-ray Photoelectron Spectroscopy
XRD: X-ray Diffraction.
