# Supplementary material for: DC Magnetron-Generated Reactive Glow Discharge Plasma Activation of Acid-Etched Titanium for Superhydrophilic TiO2‑Rich Surfaces
Source: ACS Omega. 2026 Oct 21;11(43):66633–50. doi: 10.1021/acsomega.6c06100 (PMC13736929; doi:10.1021/acsomega.6c06100)
Supplement: Supplementary file 1 [file ao6c06100_si_001.pdf]

Supporting Information

DC Magnetron-Generated Reactive Glow Discharge Plasma Activation of Acid-Etched Titanium for Superhydrophilic TiO_2-Rich Surfaces

Mickael Melo Linkiewicz[a,†,*], *Angelika von Schmude*[b,‡], *Matheus H. L. de Lima*[c], *Anderson R. Carvalho*[d], *Marcel F. Kunrath*[a,e,f], *Marcus Seferin*[g,$], *and Roberto Hübler*[a,¶]

[a] Materials and Nanoscience Laboratory, Interdisciplinary Center for Nanoscience and Micro-Nanotechnology (NanoPUCRS), Polytechnic School, PUCRS, 91530-001, Porto Alegre, RS, Brazil.

[b] Institute of Physics, UFRGS, 91501-970, Porto Alegre, RS, Brazil.

[c] Interdisciplinary Center for Nanoscience and Micro-Nanotechnology (NanoPUCRS), Polytechnic School, PUCRS, 91530-001, Porto Alegre, Rio Grande do Sul, Brazil.

[d] Chemometrics and Analytical Instrumentation Laboratory, Institute of Chemistry, UFRGS, 91501-970, Porto Alegre, Rio Grande do Sul, Brazil.

[e] Department of Dentistry, School of Health and Life Sciences, PUCRS, 90619-900, Porto Alegre, Rio Grande do Sul, Brazil.

[f] Department of Biomaterials, Institute of Clinical Sciences, Sahlgrenska Academy at the University of Gothenburg, 40530, Göteborg, Sweden.

[g] Industrial Chemistry Laboratory, Polytechnic School, PUCRS, 90619-900, Porto Alegre, RS, Brazil.

[†] Present address for Mickael Melo Linkiewicz: Materials Laboratory, Metallic Materials Engineering Nucleus, Polytechnic School, Pontifical Catholic University of Rio Grande do Sul (PUCRS), 90619-900, Porto Alegre, RS, Brazil.

[‡] Present address for Angelika von Schmude: Independent Researcher, Porto Alegre, RS, Brazil.

[§] Present address for Marcus Seferin: Chemometrics and Analytical Instrumentation Laboratory, Institute of Chemistry, Federal University of Rio Grande do Sul (UFRGS), 91501-970, Porto Alegre, RS, Brazil.

[¶] Present address for Roberto Hübler: Independent Researcher, Porto Alegre, RS, Brazil.

The surface chemical composition of all four sample conditions was evaluated by X-ray photoelectron spectroscopy (XPS) using a single sequentially treated disk analyzed under the 1CC, 2AE, 3RP, and 3RP-A conditions. Each acquisition was performed after approximately 48 h of vacuum residence. The survey spectra (**Figure 8A** of the main manuscript) showed *C 1s*, *O 1s*, and *Ti 2p* signals for all conditions, as expected from adventitious carbon, surface oxygen species, and the titanium substrate. N 1s contributions were detected for 1CC and 3RP-A (**Figure S1**), whereas an *F 1s* signal was detected only in 2AE (**Figure S2**). No *F 1s* signal was resolved for the 3RP condition under the acquisition conditions applied.

In the *Ti 2p* region (**Figure S3**), all conditions showed TiO_2-related components at approximately 458.7 and 464.4 eV. The 1CC condition also contained components assigned to titanium oxynitride species, *TiON* and *TiNO*, which together accounted for 21.4 % of the fitted *Ti 2p* area. The 2AE condition exhibited *Ti–F*-related components, along with a Ti^0 contribution of 18.6 %, consistent with exposure of the metallic substrate following *HF* etching. In 3RP, the fitted envelope was accounted for entirely by TiO_2-related components (100 %), compared with 78.6 % for 1CC and 71.5 % for 2AE. In the aged reactive plasma condition (3RP-A), the TiO_2 contribution decreased to 55.1 %, while oxynitride-related components accounted for the remaining 45.0 %. Because the 3RP-A condition included an intervening sequence of cleaning, desiccator-storage, and vacuum-equilibration, the differences between 3RP-A and 3RP cannot be attributed exclusively to ambient atmospheric aging.

The *O 1s* region (**Figure S4**) showed treatment-dependent changes in oxygen bonding environments. The component near 530.2 eV, assigned primarily to lattice *Ti–O* bonds, increased from 69.9 % for 1CC to 75.6 % for 2AE and 84.7 % for 3RP, decreasing to 46.4 % for 3RP-A. Because *O–N* and *O–H* species have overlapping *O 1s* binding-energy ranges near 531.5 eV, they were fitted as a single unresolved component, with relative area was 12.7 %, 20.1 %, 10.7 %, and 23.7 % for 1CC, 2AE, 3RP, and 3RP-A, respectively. In 1CC and 3RP-A, the corresponding *N 1s* and *Ti 2p* results support the presence of an *O–N* contribution within this envelope, whereas in 2AE and 3RP, the absence of a resolved *N 1s* signal makes an *O–H* contribution chemically plausible. The relative area of this component did not vary monotonically with the contact angle across 1CC, 2AE, and 3RP and was therefore not used as a

quantitative proxy for surface hydroxyl density or as the basis for an inferential structure–wettability correlation. The component near 532.5 eV was assigned to adsorbed water and oxygen-containing organic species. Its relative area decreased from 17.4 % for 1CC to 4.3 % for 2AE and 4.7 % for 3RP, before increasing to 29.9 % for 3RP-A.

The *N 1s*, *F 1s*, *Ti 2p*, and *O 1s* regions were curve-fitted, and the resulting binding energies, full widths at half maximum (FWHM), and relative contributions are summarized in **Table S1**. The *C 1s* region showed no relevant treatment-dependent differences and was assigned mainly to adventitious carbon, it was therefore not curve-fitted; no *C 1s* parameters are reported.

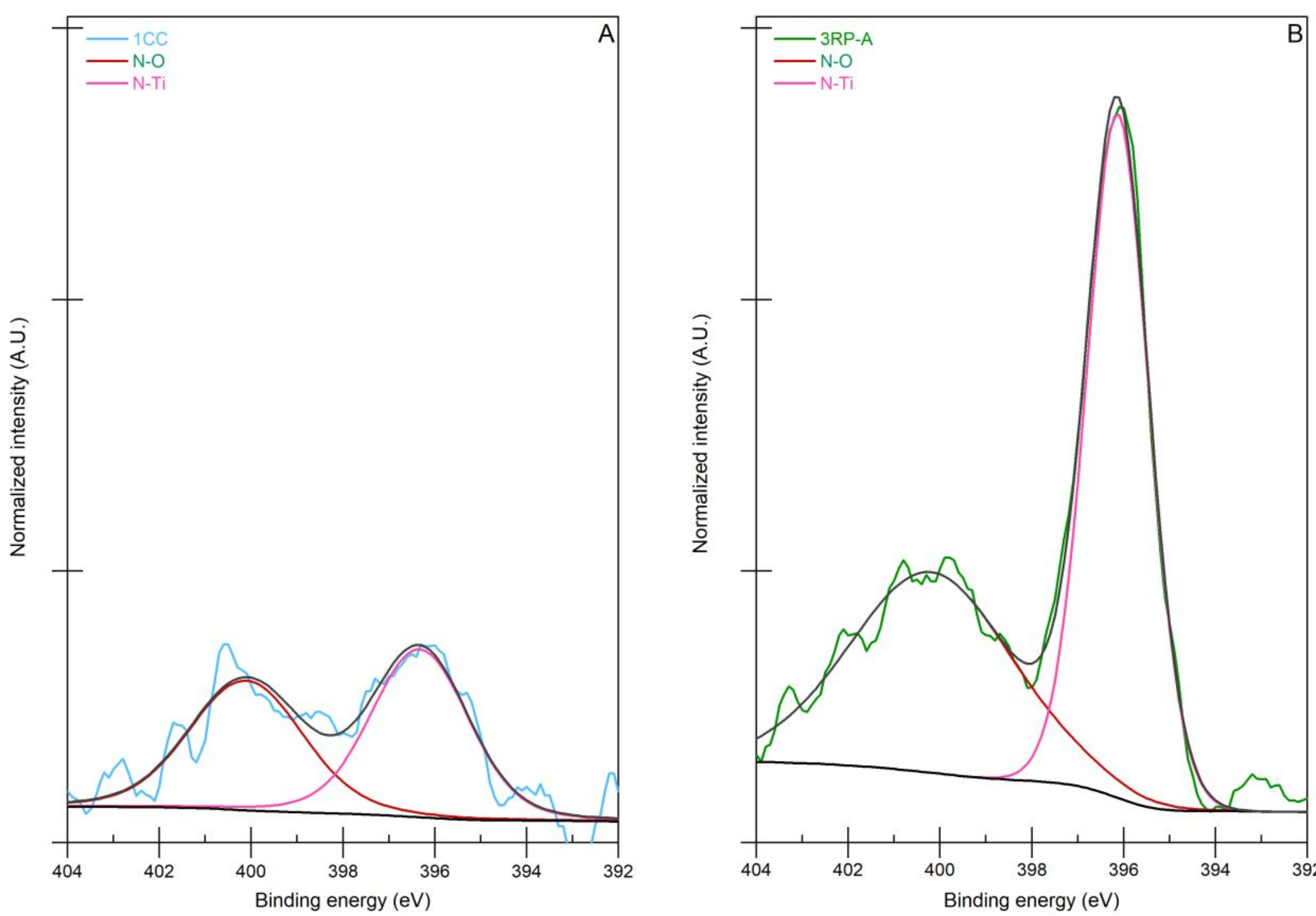

Figure S1. Expanded high-resolution *N 1s* core-level XPS spectra for (A) 1CC and (B) 3RP-A samples, complementing Figure 8B in the main manuscript. No *N 1s* signal was resolved for 2AE or 3RP.

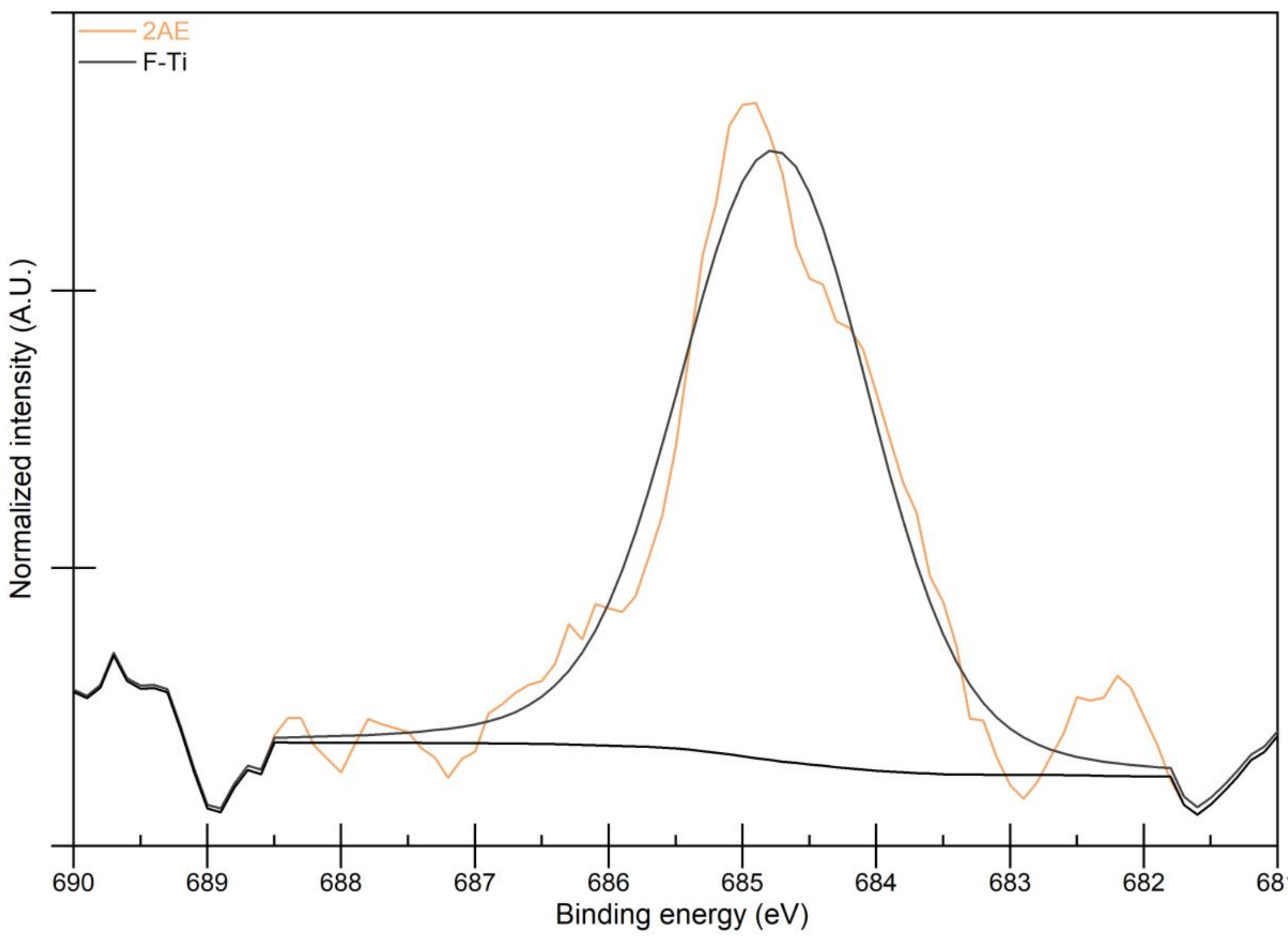

Figure S2. Expanded high-resolution *F 1s* core-level XPS spectrum for the 2AE sample, complementing Figure 8C in the main manuscript. No *F 1s* signal was resolved for 3RP.

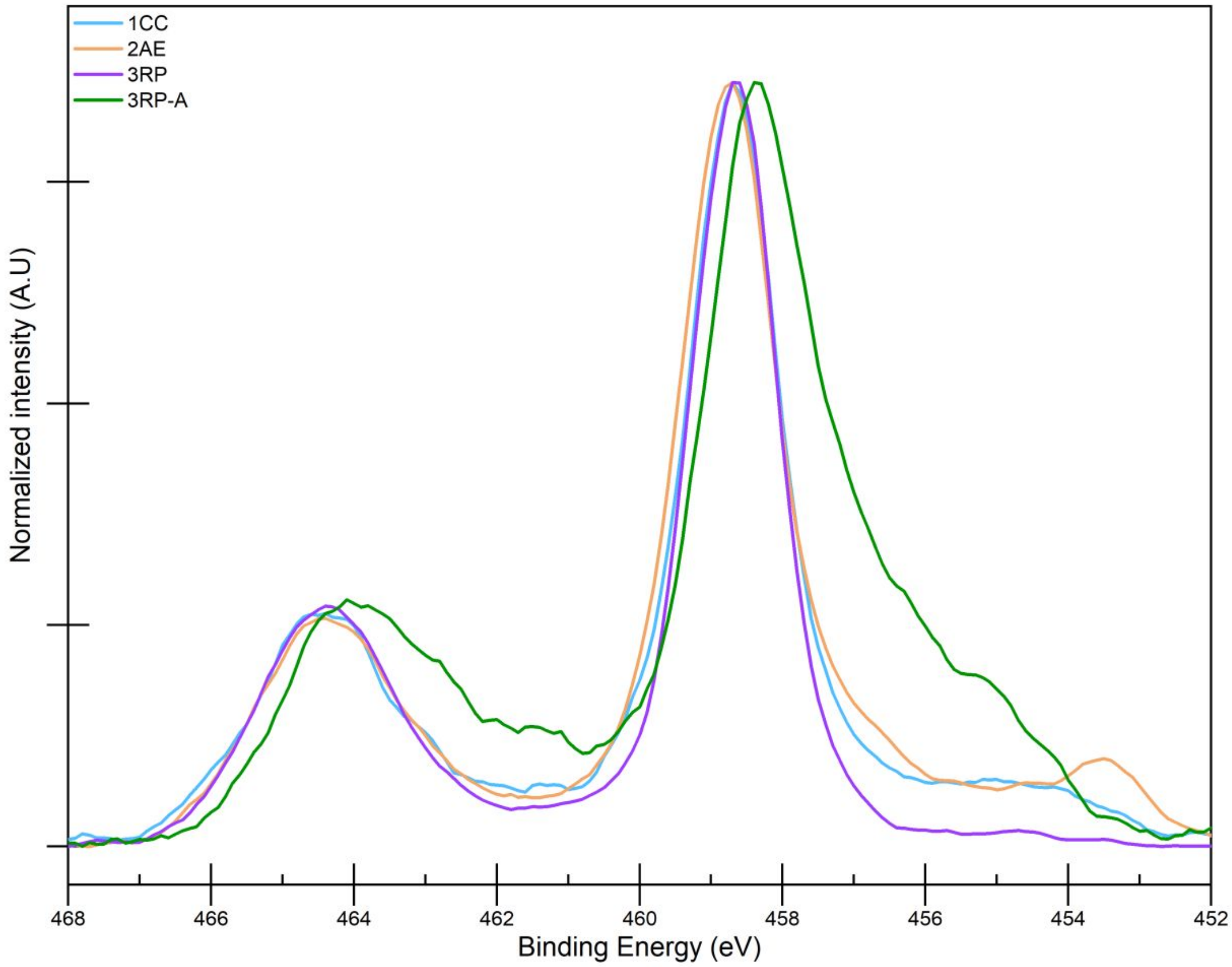

Figure S3. High-resolution *Ti 2p* core-level XPS spectra of titanium samples subjected to 1CC, 2AE, 3RP, and 3RP-A. The fitting parameters are provided in **Table S1**.

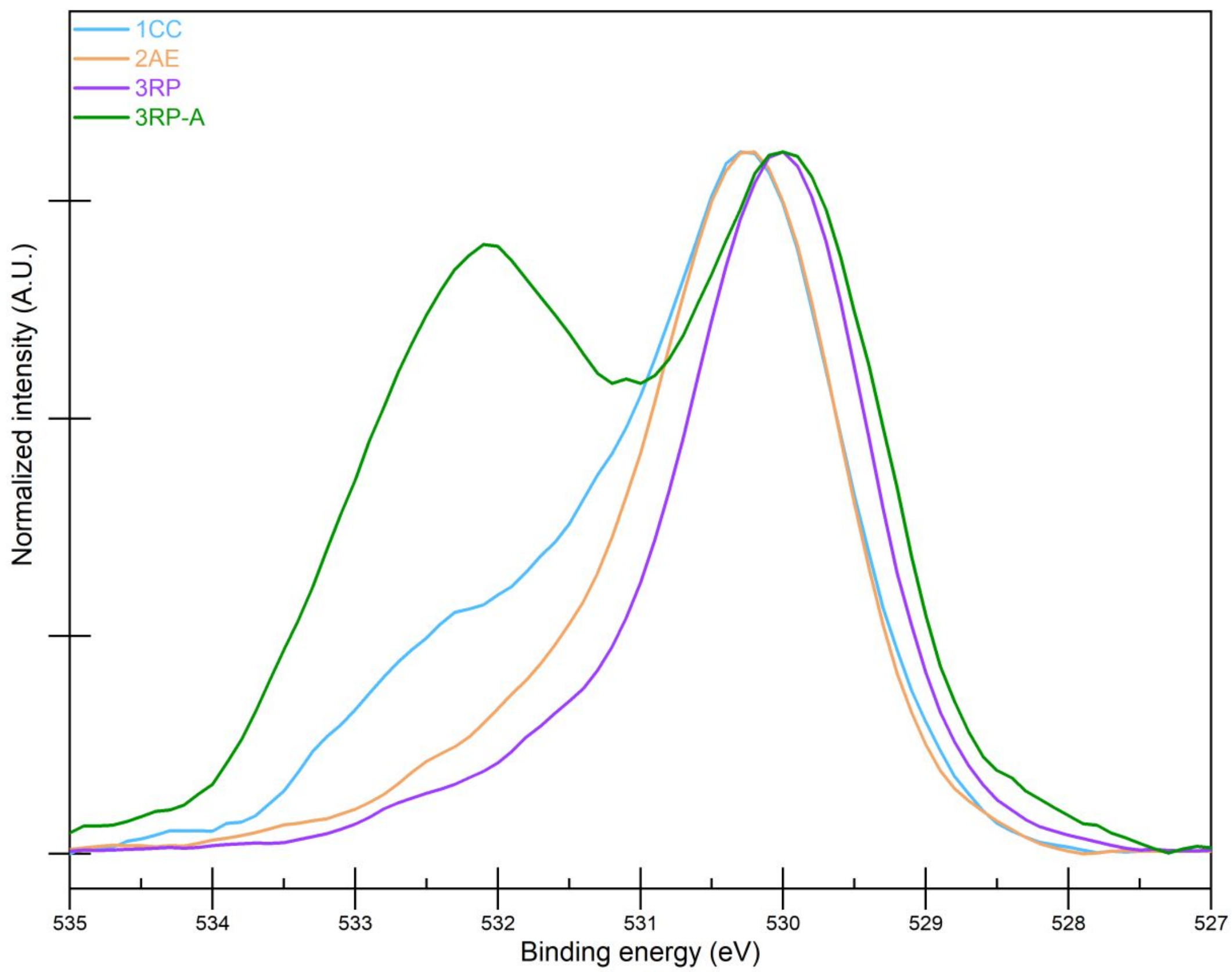

Figure S4. High-resolution *O 1s* core-level XPS spectra of 1CC, 2AE, 3RP, and 3RP-A, showing treatment-dependent oxygen bonding environments. The fitting parameters are provided in **Table S1**.

Table S1. XPS curve-fitting parameters for the high-resolution *N 1s*, *F 1s*, *Ti 2p*, and *O 1s* regions of titanium samples after different surface treatments.

Core level	Component assignment	Sample	B.E. main peak (eV)	FWHM (eV)	Relative peak-area contribution (%)
N 1s	*N-Ti*	1CC	396.33	2.50	52.7
N 1s	*N-O*	1CC	400.11	2.91	47.3
N 1s	*N-Ti*	3RP-A	396.13	1.60	57.9
N 1s	*N-O*	3RP-A	400.20	4.18	42.1
F 1s	*Ti-F*	2AE	684.76	1.68	100.0
Ti 2p	Ti^0	2AE	453.47 / 459.56	1.72 / 1.80	18.6
Ti 2p	TiO_2	1CC	458.68 / 464.38	1.50 / 2.39	78.6
Ti 2p	TiO_2	2AE	458.75 / 464.45	1.54 / 2.41	71.5
Ti 2p	TiO_2	3RP	458.68 / 464.38	1.48 / 2.39	100.0
Ti 2p	TiO_2	3RP-A	458.50 / 464.20	1.57 / 2.19	55.1
Ti 2p	*TiON*-related	1CC	456.63 / 461.83	2.15 / 2.30	11.8
Ti 2p	Ti-F-related	2AE	456.91 / 462.12	2.30 / 2.50	9.9
Ti 2p	*TiON*-related	3RP-A	457.14 / 462.34	1.99 / 2.30	29.9
Ti 2p	*TiNO*-related	1CC	454.39 / 459.99	2.00 / 2.20	9.6
Ti 2p	*TiNO*-related	3RP-A	455.09 / 460.70	2.00 / 2.20	15.1
O 1s	*Ti-O* lattice oxygen	1CC	530.23	1.52	69.9
O 1s	*Ti-O* lattice oxygen	2AE	530.20	1.42	75.6
O 1s	*Ti-O* lattice oxygen	3RP	530.02	1.44	84.7
O 1s	*Ti-O* lattice oxygen	3RP-A	529.95	1.59	46.4
O 1s	*O-N / O-H (unresolved)*	1CC	531.48	1.16	12.7
O 1s	*O-N / O-H (unresolved)*	2AE	531.46	1.85	20.1
O 1s	*O-N / O-H (unresolved)*	3RP	531.45	1.39	10.7
O 1s	*O-N / O-H (unresolved)*	3RP-A	531.78	1.75	23.7
O 1s	Adsorbed H_2O / oxygen-containing organic species	1CC	532.50	1.44	17.4
O 1s	Adsorbed H_2O / oxygen-containing organic species	2AE	532.50	2.20	4.3
O 1s	Adsorbed H_2O / oxygen-containing organic species	3RP	532.50	1.54	4.7
O 1s	Adsorbed H_2O / oxygen-containing organic species	3RP-A	532.50	1.98	29.9

Notes:

(1) B.E., binding energy; FWHM, full width at half maximum. For *Ti 2p* components, paired B.E. and FWHM values correspond to $Ti\ 2p_{3/2}$ and $Ti\ 2p_{1/2}$, respectively, and the reported relative contribution was calculated from the summed area of each spin-orbit doublet. For *N 1s*, *F 1s*, and *O 1s*, relative peak-area contributions were calculated within each fitted core-level region. These values are relative contributions within each region and do not represent overall surface atomic concentrations.

(2) For the 3RP condition, no resolved *F 1s* signal was detected within the sensitivity of the measurement; therefore, no *F 1s* fitting parameters are reported for this condition. Components absent under a given condition are omitted from the table rather than reported as zero.

(3) The *O 1s* component near 531.5 eV represents an unresolved envelope containing potentially overlapping *O–N* and *O–H* contributions. The reported relative areas must not be interpreted as separate or exclusive *O–N* or *O–H* fractions. Treatment-dependent interpretation was based indirectly on complementary *N 1s* and *Ti 2p* results.

(4) No inferential correlation was calculated between the relative area of the unresolved *O–N/O–H* component and the water contact angle. The XPS areas were obtained from one sequentially treated disk, whereas contact angles were measured on independent disks (n = 60 for 1CC, n = 60 for 2AE, and n = 20 for 3RP). Descriptive comparison yielded *O–N/O–H* contributions/contact angles of 12.7%/47.1 ± 4.3°, 20.1%/23.2 ± 6.6°, and 10.7%/13.5 ± 1.0° for 1CC, 2AE, and 3RP,

respectively. The nonmonotonic relationship and unresolved nature of this component preclude its interpretation as a quantitative measure of surface hydroxylation.

(5) FWHM values were allowed to vary freely between conditions during fitting. The N 1s components reported for 1CC and 3RP-A therefore represent envelopes comprising multiple nitrogen bonding environments rather than single chemical states, which accounts for the differences in width between the two conditions.

REFERENCES

1. Kongsong, P.; Hasook, C.; Changpru, C.; Sangchay, W.; Konkhunthot, N. Effect of Different Chemical Etching Solutions on Physical and Chemical Surface Properties of Commercially Pure Titanium Grade 2. *J. Mater. Eng. Perform.* **2023**, 32, 5060–5071. DOI: 10.1007/s11665-022-07442-8

2. Becker, S.; Merz, R.; Hasse, H.; Kopnarski, M. Solvent Cleaning and Wettability of Technical Steel and Titanium Surfaces. *Adsorpt. Sci. Technol.* **2016**, 34, 509–524. DOI: 10.1177/0263617416645110

3. Martins, G. M.; Silva Braz, J. K. F.; Macedo, M. F.; Vitoriano, J. O.; Alves Júnior, C.; Santos, C. S.; Feijó, F. M. C.; Oliveira, M. F.; Moura, C. E. B. Enhancing Titanium Disk Performance Through In-Pack Cold Atmospheric Plasma Treatment. *ACS Biomater. Sci. Eng.* **2024**, 10, 1765–1773. DOI: 10.1021/acsbiomaterials.3c01388

4. Jung, A.; Lee, H.; Kim, H.; Jeon, H. J.; Park, S.; Gweon, B. Impact of Plasma Discharge Pressure on Implant Surface Properties and Osteoblast Activities in Vacuum-Assisted Plasma Treatment. *Sci. Rep.* **2024**, 14, 31757. DOI: 10.1038/s41598-024-82730-y

5. Jeon, H.-J.; Seo, S.; Jung, A.; Kang, K.-m.; Lee, J.; Gweon, B.; Lim, Y. Comparative Investigation of Vortex and Direct Plasma Discharge for Treating Titanium Surface. *Biomimetics* **2025**, 10, 7. DOI: 10.3390/biomimetics10010007

6. Katz, J.; Gershman, S.; Belkind, A. Optical Emission Spectroscopy and Contact Angle Study of Plasma Cleaning of Titanium Alloy Surfaces: Argon Plasma. *Plasma Med.* **2015**, 5, 223–236. DOI: 10.1615/PlasmaMed.2016015722

7. Vitoriano, J. O.; de Moura, C. E. B.; Braz, J. F. S.; Martins, G. M.; Barros, P. P.; Santos, C. S.; Melo, R. L. F.; do Carmo, F. R.; Pessoa, R. S.; Alves-Junior, C. Aging Behavior of Titanium Surfaces Treated with Non-Thermal Atmospheric Pressure Plasma at Different Temperatures. Appl. Surf. Sci. **2026**, 715, 164537. DOI: 10.1016/j.apsusc.2025.164537

8. Choi, S.-H.; Ryu, J.-H.; Kwon, J.-S.; Kim, J.-E.; Cha, J.-Y.; Lee, K.-J.; Yu, H.-S.; Choi, E.-H.; Kim, K.-M.; Hwang, C.-J. Effect of Wet Storage on the Bioactivity of Ultraviolet Light- and Non-Thermal Atmospheric Pressure Plasma-Treated Titanium and Zirconia Implant Surfaces. Mater. Sci. Eng. C **2019**, 105, 110049. DOI: 10.1016/j.msec.2019.110049

9. Popov, A. A.; Tikhonowski, G. V.; Shakhov, P. V.; Popova-Kuznetsova, E. A.; Tselikov, G. I.; Romanov, R. I.; Markeev, A. M.; Klimentov, S. M.; Kabashin, A. V. Synthesis of Titanium Nitride Nanoparticles by Pulsed Laser Ablation in Different Aqueous and Organic Solutions. *Nanomaterials* **2022**, 12, 1672. DOI: 10.3390/nano12101672

10. Kumaresan, L.; Amir, H.; Shanmugavelayutham, G.; Viswanathan, C. Plasma Assists Titanium Nitride and Surface Modified Titanium Nitride Nanoparticles from Titanium Scraps for Magnetic Properties and Supercapacitor Applications. *Ceram. Int.* **2022**, 48, 30393–30406. DOI: 10.1016/j.ceramint.2022.06.317

11. Maarouf, M.; Haider, M. B.; Drmosh, Q. A.; Mekki, M. B. X-Ray Photoelectron Spectroscopy Depth Profiling of As-Grown and Annealed Titanium Nitride Thin Films. *Crystals* **2021**, 11, 239. DOI: 10.3390/cryst11030239

12. Hsu, J.-C.; Lin, Y.-H.; Wang, P. W. X-ray Photoelectron Spectroscopy Analysis of Nitrogen-Doped TiO_2 Films Prepared by Reactive-Ion-Beam Sputtering with Various NH_3/O_2 Gas Mixture Ratios. *Coatings* **2020**, 10, 47. DOI: 10.3390/coatings10010047

13. Korotin, D. M.; Bartkowski, S.; Kurmaev, E. Z.; Neumann, M.; Yakushina, E. B.; Valiev, R. Z.; Cholakh, S. O. Surface Studies of Coarse-Grained and Nanostructured Titanium Implants. *J. Nanosci. Nanotechnol.* **2012**, 12, 8567–8572. DOI: 10.1166/jnn.2012.6806

14. Korotin, D. M.; Bartkowski, S.; Kurmaev, E. Z.; Meumann, M.; Yakushina, E. B.; Valiev, R. Z.; Cholakh, S. O. Surface Characterization of Titanium Implants Treated in Hydrofluoric Acid. *J. Biomater. Nanobiotechnol.* **2012**, 3, 87–91. DOI: 10.4236/jbnb.2012.31011

15. Stájer, A.; Ungvári, K.; Pelsőczi, I. K.; Polyánka, H.; Oszkó, A.; Mihalik, E.; Rakonczay, Z.; Radnai, M.; Kemény, L.; Fazekas, A.; Turzó, K. Corrosive Effects of Fluoride on Titanium: Investigation by X-ray Photoelectron Spectroscopy, Atomic Force Microscopy, and Human

Epithelial Cell Culturing. *J. Biomed. Mater. Res. A* **2008**, 87A, 450–458. DOI: 10.1002/jbm.a.31764

16. Lv, Z.; Wang, L.; Hu, X.; Bu, Z.; Li, Y. Leveling Mechanism of Plasma Electrolytic Polishing of Titanium Alloy with Aqueous Fluoride-Type Solution. *Int. J. Electrochem. Sci.* **2024**, 19, 100749. DOI: 10.1016/j.ijoes.2024.100749

17. Wang, L.; Wang, M.; Zhong, M.; Li, X.; Cui, Z. Degradation of Thermal Oxide Film on Pure Titanium in an Acidic Environment Containing Fluoride. *NPJ Mater. Degrad.* **2022**, 6, 65. DOI: 10.1038/s41529-022-00275-9

18. Eid, K.; Sliem, M. H.; Abdullah, A. M. Tailoring the Defects of Sub-100 nm Multipodal Titanium Nitride/Oxynitride Nanotubes for Efficient Water Splitting Performance. *Nanoscale Adv.* **2021**, 3, 5016–5026. DOI: 10.1039/D1NA00274K

19. Chang, C.-C.; Nogan, J.; Yang, Z.-P.; Kort-Kamp, W. J. M.; Ross, W.; Luk, T. S.; Dalvit, D. A. R.; Azad, A. K.; Chen, H.-T. Highly Plasmonic Titanium Nitride by Room-Temperature Sputtering. *Sci. Rep.* **2019**, 9, 15287. DOI: 10.1038/s41598-019-51236-3
